# Brain Endothelial Cells Control Fertility through Ovarian-Steroid–Dependent Release of Semaphorin 3A

**DOI:** 10.1371/journal.pbio.1001808

**Published:** 2014-03-11

**Authors:** Paolo Giacobini, Jyoti Parkash, Céline Campagne, Andrea Messina, Filippo Casoni, Charlotte Vanacker, Fanny Langlet, Barbara Hobo, Gabriella Cagnoni, Sarah Gallet, Naresh Kumar Hanchate, Danièle Mazur, Masahiko Taniguchi, Massimiliano Mazzone, Joost Verhaagen, Philippe Ciofi, Sébastien G. Bouret, Luca Tamagnone, Vincent Prevot

**Affiliations:** 1 INSERM, Jean-Pierre Aubert Research Center, U837, Development and Plasticity of the Postnatal Brain, Lille, France; 2 UDSL, School of Medicine, Place de Verdun, Lille, France; 3 Institut de Médecine Prédictive et de Recherche Thérapeutique, IFR114, Lille, France; 4 Netherlands institute for Neuroscience, Amsterdam, The Netherlands; 5 Center for Neurogenomics and Cognitive Research, Vrije Universiteit Amsterdam, Amsterdam, The Netherlands; 6 Candiolo Cancer Institute - FPO (IRCCS) and University of Torino, Department of Oncology, Candiolo, Italy; 7 Research Institute for Frontier Medicine, Sapporo Medical University School of Medicine, Sapporo, Japan; 8 Versalius Research Center, VIB, Laboratory of Molecular Oncology and Angiogenesis, Leuven, Belgium; 9 KU Keuven, Versalius Research Center, Leuven, Belgium; 10 INSERM, Neurocentre Magendie, U862, Université de Bordeaux, Bordeaux, France; 11 The Saban Research Institute, Childrens Hospital Los Angeles, University of Southern California, Los Angeles, California, United States of America; University of Cambridge, United Kingdom

## Abstract

Neuropilin-1 (Nrp1) guides the development of the nervous and vascular systems, but its role in the mature brain remains to be explored. Here we report that the expression of the 65 kDa isoform of Sema3A, the ligand of Nrp1, by adult vascular endothelial cells, is regulated during the ovarian cycle and promotes axonal sprouting in hypothalamic neurons secreting gonadotropin-releasing hormone (GnRH), the neuropeptide controlling reproduction. Both the inhibition of Sema3A/Nrp1 signaling and the conditional deletion of Nrp1 in GnRH neurons counteract Sema3A-induced axonal sprouting. Furthermore, the localized intracerebral infusion of Nrp1- or Sema3A-neutralizing antibodies *in vivo* disrupts the ovarian cycle. Finally, the selective neutralization of endothelial-cell Sema3A signaling in adult *Sema3a* ^loxP/loxP^ mice by the intravenous injection of the recombinant TAT-Cre protein alters the amplitude of the preovulatory luteinizing hormone surge, likely by perturbing GnRH release into the hypothalamo-hypophyseal portal system. Our results identify a previously unknown function for 65 kDa Sema3A-Nrp1 signaling in the induction of axonal growth, and raise the possibility that endothelial cells actively participate in synaptic plasticity in specific functional domains of the adult central nervous system, thus controlling key physiological functions such as reproduction.

## Abstract 2

Endothelial-cell–derived Sema3A promotes axonal outgrowth and plasticity and thereby regulates neurohormone release in the adult rodent brain in response to the ovarian cycle.

## Author Summary

In the developing embryo, endothelial cells release chemotropic signals such as Semaphorin 3A (Sema3A) that, upon activation of its receptor Neuropilin-1 (Nrp1), regulate neuronal migration and axon guidance. However, whether endothelial cells in the adult brain retain the ability to secrete molecules that influence neuronal function is unknown. Here we show in the adult brain of rodents that vascular endothelial cells release Sema3A and that the amount released is regulated by the ovulatory cycle. Sema3A, in turn, promotes the outgrowth of axons of hypothalamic neurons that express Neuropilin-1 towards the endothelial wall of portal blood vessels. These neurons release there the neuropeptide that controls reproduction: gonadotropin-releasing hormone (GnRH). Notably, this endothelial-cell-mediated sprouting of GnRH axons regulates neuropeptide release at a key stage of the estrous cycle, the proestrus, when the surge of GnRH triggers ovulation. Thus, by promoting GnRH axonal growth in the adult brain, Sema3A/Neuropilin-1 plays a pivotal role in orchestrating the central control of reproduction. Our results suggest a model in which vascular endothelial cells are dynamic signaling components that relay peripheral information to the brain to control key physiological functions, including species survival.

## Introduction

**Figure 1:**
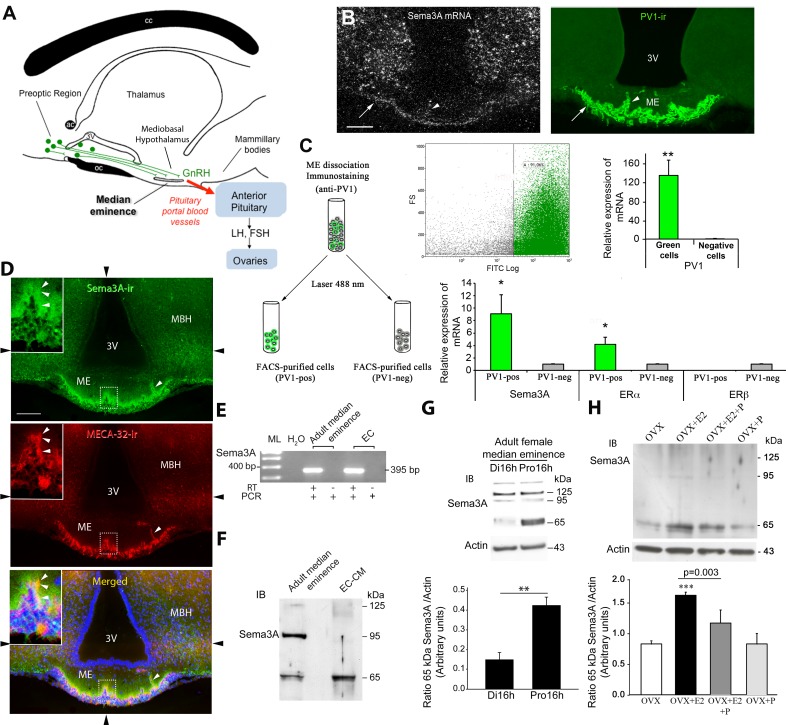
Sema3A expression in ME vascular endothelial cells during the ovarian cycle. (A) Schematic diagram illustrating the anatomy of the hypothalamic-pituitary-gonadal axis in a sagittal view. In rodents, GnRH cell bodies (green circles) are diffusely distributed in the preoptic region and send neuroendocrine axons (green fibers) towards the ME of the hypothalamus, where they release the neurohormone into pituitary portal blood vessels (red arrow) for delivery to the anterior pituitary. At the adenohypophysis, GnRH elicits the secretion of the gonadotropins luteinizing hormone (LH) and follicle-stimulating hormone (FSH), which stimulate gametogenesis and gonadal-steroid secretion and thus support reproductive function. cc, corpus calosum; ac, anterior comissure; oc, optic chiasma; 3V, third ventricle. (B) Representative dark-field photomicrographs of a coronal section of an adult female rat ME, showing Sema3A mRNA localized using a radioactive probe (bright dots indicating silver grains, top panel). Note the presence of Sema3A mRNA in the capillary zone of the ME (white arrow) and in intrainfundibular capillary loops (arrowhead) containing PV1-immunoreactive fenestrated endothelial cells (right panel, green immunofluorescence), and its relative paucity in the parenchyma. Sema3A mRNA expression is also seen in various nuclei of the mediobasal hypothalamus (MBH) that lie adjacent to the ME but do not contain PV1-immunoreactive blood vessels. V3, third ventricle. Scale bar, 100 µm. (C) PV1-positive cell (PV1-pos) isolation by FACS (schematic diagram and dot plot, top) and real-time PCR analysis of PV1, Sema3A, estrogen receptor alpha (ERα), and ERβ transcripts. (D) Representative immunofluorescence images showing the localization of Sema3A immunoreactivity (green) in coronal sections of the ME of adult female mice. Fenestrated vascular endothelial cells are labeled by the monoclonal antibody MECA32, which binds to mouse PV1 (red). Note that Sema3A immunoreactivity is localized in portal blood capillaries of the external zone of the ME (inset) as well as some intrainfundibular capillary loops present in the nervous parenchyma (arrowhead); Sema3A immunolabelling is of very high intensity at the level of the capillary zone, but is also seen in the adjacent nervous parenchyma, progressively vanishing at deeper levels of the tissue. Nuclei are counterstained in blue using Hoechst. (Insets) High-magnification images of the areas indicated by dashed lines. Black arrowheads at the periphery of the pictures indicate the planes of the individual images that make up the photomontage; each panel is composed of an assembly of four images captured sequentially for each fluorophore using the MosaiX module of the AxioVision 4.6 system (Zeiss, Germany) and a Zeiss 20× objective (N.A. 0.8). Scale bar, 100 µm (30 µm in inset). (E) Detection by RT-PCR of Sema3A mRNA in total RNA extracts from ME explants microdissected from adult female rats and immunopurified ME endothelial cells (ECs). ML, 100 bp molecular ladder; H_2_O, PCR negative control without cDNA; +/−, Sema3A amplicon (395 bp) with (+) or without (−) RT. (F) Western blot analysis of Sema3A protein levels in the adult ME and 48 h ME EC-conditioned medium (EC-CM). Each lane was loaded with 35 µg of protein. While all Sema3A isoforms (65, 95, and 125 kDa) are detected in protein extracts from the adult female ME, only the 65 kDa Sema3A isoform is present in the EC-CM. (G) Western blot (top) and quantitative analysis (bottom, relative to actin) of Sema3A protein levels showing a difference in 65 kDa Sema3A expression between the afternoon of diestrus (Di16h) and proestrus (Pro16h), whereas the protein levels of 125 and 95 kDa Sema3A remain unchanged. Band intensity was quantified using Scion software. ** t_(8)_ = 4.709, *p* = 0.0015 (*n* = 5 independent experiments). (H) Western blot analysis for Sema3A (top; upper image) and actin (top; lower image) in the ME of control ovariectomized (OVX) female rats and those treated with 17β-estradiol 3-benzoate (E2), progesterone (P), or E2+P (*n* = 5 independent experiments per treatment). E2 induces a significant increase in 65 kDa Sema3A expression in the ME of OVX rats when compared with the other treatment groups (one-way ANOVA, F_(8,11)_ = 27.779, *p*<0.001; Tukey's test, ***p<0.001), whereas progesterone inhibits this increase (Tukey's test, *p* = 0.003). Bar graph, mean ratio (±SEM) of Sema3A expression to that of actin (*p*<0.05, one-way ANOVA). Data are represented as means ± SEM.

Blood vessels and axons employ similar mechanisms and follow common guidance cues to grow and navigate tissues during embryonic development [1],[2]. Blood vessels influence the trajectories taken by axons to reach their appropriate end organs [3]. In the adult brain, they communicate with neurons and glia in order to meet physiological demands [4],[5]. Endothelial cells are well positioned to sense peripheral inputs and ideally suited to convey signals that could influence neuronal structure and synaptic plasticity. However, whether they are capable of influencing axonal plasticity in the mature central nervous system remains to be elucidated. Recent evidence suggests that the semaphorins, members of a family of secreted guidance molecules, continue to be expressed in the postnatal brain and may have important implications for neuronal plasticity and nervous system physiology [6]. Of these, Sema3A, which exerts both repulsive and attractive effects on growing axons [7]–[9], is also expressed in endothelial cells during vascular development [10],[11]. Interestingly, Sema3A acts as a guidance factor during the migration of a particular population of neuroendocrine neurons that secrete the fertility-regulating neuropeptide gonadotropin-releasing hormone (GnRH) [12],[13], and that moreover retain a high degree of plasticity in the mature brain [14]. In particular, GnRH neurons, which project to the hypothalamic median eminence (ME) and release their neurohormone into a specialized capillary network for delivery to the anterior pituitary (Figure 1A), are known to undergo extensive axonal growth towards the vascular wall during critical time windows in adulthood, such as at the onset of the preovulatory surge, when massive GnRH release has to occur to trigger ovulation [14], and are thus an ideal system in which to analyze endothelial-axonal interactions during adult nervous system homeostasis.

In this study, we examined whether this periodic sprouting of GnRH axon terminals in the ME of the adult hypothalamus was regulated by endothelial cells, through the release of Sema3A and the activation of its cognate receptor, neuropilin-1 (Nrp1) [15]–[17]. We report that endothelial cells of the ME do indeed release the 65 kDa isoform of Sema3A (p65-Sema3A) at key stages of the ovarian cycle, that Nrp1 is expressed in GnRH axons, and that Sema3A-Nrp1 signaling is required for the extension of GnRH axon terminals towards the vascular plexus on the day of the preovulatory surge. We also demonstrate that the selective inhibition of Sema3A expression in endothelial cells of the ME and the transient local manipulation of Sema3A signaling *in vivo* alter the preovulatory release of GnRH, suggesting that the endothelium-to-neuron communication mediated by 65 kDa Sema3A-Nrp1 signaling is of functional relevance in the adult brain. Our results thus indicate a hitherto unidentified role for brain vascular endothelial cells in mediating the cyclic plasticity of GnRH axons in the adult hypothalamus and, consequently, in reproductive physiology.

## Results

### Sema3A Is Expressed by the Endothelial Cells of Portal Blood Vessels in the ME of the Adult Hypothalamus

Sema3A is mainly known as a developmental signal regulating axon guidance. In order to assess the potential role of Sema3A as a guidance cue for hypothalamic GnRH neurons controlling the ovarian cycle, we first investigated its expression in the ME of adult animals. In situ hybridization of adult female rat brain sections revealed that the mRNA for Sema3A was selectively expressed in endothelial cells of the vascular compartment of the ME (Figure 1B). Only a weak hybridization signal was seen in the ependymal layer and in the internal and external axon layers. Brain sections hybridized with the sense probe (negative control) did not exhibit any detectable labeling in the ME (unpublished data). Further analysis by cell sorting, using an affinity-purified antibody to plasmalemmal vesicle-associated protein 1 (PV1) [18], a component of the fenestral diaphragms [19], selectively expressed by endothelial cells of the ME (Figure 1B,C; PV1 mRNA expression in fluorescent versus nonfluorescent cells, t_(6)_ = 4.080, *p* = 0.007, *n* = 4) revealed that Sema3A expression was restricted to PV1-positive cells (Figure S1 and Figure 1C; t_(6)_ = 2.636, *p* = 0.039, *n* = 4), unlike β3-tubulin, DARPP-32, and thyroid-stimulating hormone (TSH), markers for neurons, tanycytes, and endocrine cells, respectively, which were expressed only by non-PV1-positive cells and not found in the same fraction as Sema3A (Figure S1). Immunofluorescence analysis in adult female mice using a Sema3A-specific antibody [13] revealed bright Sema3A immunoreactivity in the capillary zone of the ME that extended into the nervous tissue, where it progressively vanished (Figure 1D). Together, these findings indicate that Sema3A is expressed *in vivo* in the ME of the mature brain, and is localized in vascular endothelial cells of the pituitary portal system, onto which GnRH neurons abut.

### Vascular Endothelial Cells Isolated from the ME Release p65-Sema3A

To further investigate the site of origin of Sema3A in portal blood vessels and to determine whether fenestrated endothelial cells from the rat ME can release Sema3A, we used a sequential panning method for their purification, as described previously [20],[21]. Consistent with our findings *in vivo* [18],[21], purified ME endothelial cells in culture expressed PV-1 and were labeled by *Bandeiraea simplicifolia* lectin (Figure S2). We confirmed Sema3A mRNA expression in purified ME endothelial cells by RT-PCR expression analysis (Figure 1E). Next, we used immunoblotting to analyze the conditioned medium of purified ME endothelial cells and compared it with total protein extracts from ME explants, revealing several bands corresponding to the different known isoforms (the secreted 65 kDa and 95 kDa forms, and the 125 kDa precursor; see Figure 1F) of Sema3A [22]. Notably, the conditioned medium of purified ME endothelial cells only contained a smear at 125 kDa and a discrete band for p65-Sema3A (Figure 1F), which appears to be the furin cleavage product of the 95 kDa isoform (Figure S2B). This confirms that fenestrated endothelial cells of the ME express Sema3A and release its 65 kDa isoform into the extracellular space.

### p65-Sema3A Release in the ME Is Regulated by the Ovarian Cycle

To determine whether Sema3A expression in the ME varies during the ovarian cycle, we performed Western blotting experiments during the onset of the preovulatory surge at proestrus (when GnRH nerve terminals are close to portal plexus vessels) and during diestrus (when GnRH nerve terminals are distant from the endothelial wall) [23]. Remarkably, we found that p65-Sema3A expression was significantly increased on the day of proestrus as compared to diestrus (Figure 1G; p65-Sema3A, Di16h versus Pro16h; *n* = 5 independent experiments, *p*<0.01, *t* test). This regulation appeared to be selective for p65-Sema3A, as the expression of other Sema3A isoforms did not change significantly during the ovarian cycle (Figure 1G; p95-Sema3A, 0.226±0.0449 arbitrary units at Di16h versus 0.133±0.0411 arbitrary units at Pro16h, *n* = 5, t_(8)_ = 1.522, *p* = 0.167; p125-Sema3A, 0.427±0.0455 arbitrary units at Di16h versus 0.379±0.0519 arbitrary units at Pro16h, *n* = 5, t_(8)_ = 0.698, *p* = 0.505). These data indicate that the levels of p65-Sema3A in the ME are maximal on the day of proestrus, when circulating levels of estradiol are also high and are known to exert their positive feedback effect on the hypothalamo-pituitary-gonadal axis [24],[25]. To determine whether these changes are sex-steroid-dependent, we ovariectomized (OVX) adult cycling female rats and subsequently treated them with subcutaneous injections of sesame oil, alone or containing 17β-estradiol 3-benzoate (E_2_), progesterone (P), or E_2_+P. As shown in Figure 1H, estradiol induced a significant increase in p65-Sema3A expression in the ME of OVX rats when compared with the other treatment groups, whereas progesterone inhibited this increase (*n* = 5 rats per treatment, *p*<0.05, one-way ANOVA). Interestingly, additional RT-PCR analyses revealed that PV1-positive endothelial cells expressed mRNA for the estrogen receptor ERα (Figure 1C) and that this expression was particularly enriched in the ME of adult female rats (PV1-positive versus PV1-negative cells, t_(6)_ = 2.793, *p* = 0.031, *n* = 4).

Altogether, these results provide direct evidence that the release of p65-Sema3A by fenestrated endothelial cells of the ME is strictly regulated during the ovarian cycle, being maximal during proestrus, under the action of circulating estradiol.

### GnRH Neurons Express the Sema3A Receptor Nrp1

**Figure 2:**
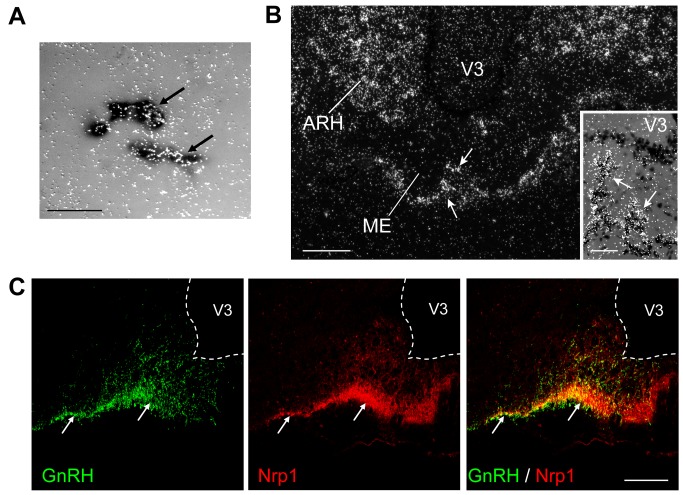
Nrp1, the obligate Sema3A receptor, is expressed in adult GnRH neurons. (A) Simultaneous bright-field and epi-illumination photomicrograph showing cells (arrows) labeled with a digoxigenin-conjugated probe for GnRH mRNA (dark staining) and a radioactive probe for Nrp1 mRNA (bright silver grains) in the preoptic region of an adult female rat. Scale bar, 20 µm. (B) Representative dark-field photomicrograph of Nrp1 mRNA (bright dots) in an adult female rat ME localized using a radioactive probe. Note the absence of signal in the parenchyma of the ME but intense expression of Nrp1 mRNA in the capillary zone of the ME (white arrows). V3, third ventricle; ARH, arcuate nucleus of the hypothalamus. Scale bar, 150 µm. (Inset) High-magnification image of a different field in the same area under simultaneous bright-field and epi-illumination to visualize cell nuclei counterstained for Nissl. Scale bar, 50 µm. (C) Photomicrographs showing the distribution of GnRH (green) and Nrp1 (red) immunoreactivity in the ME of an adult female rats. Note that Nrp1 and GnRH are colocalized in the external layer of the ME (arrows). Scale bar, 100 µm.

We next investigated whether adult GnRH neurons express Nrp1, the obligate receptor of Sema3A, by performing double *in situ* hybridization experiments using ^33^P-labeled Nrp1 and Dig-UTP-labeled GnRH antisense cRNA probes (Figure 2A). High levels of Nrp1 mRNA were observed in cells of the diagonal band of Broca (DBB) and in several nuclei of the rostral and medial preoptic regions—for example, the anteroventral periventricular nucleus, the median preoptic nucleus, and the medial preoptic nucleus (unpublished data). The distribution of neurons expressing GnRH mRNA was similar to that described in previous *in situ* hybridization studies [26]–[28]—that is, the highest density was seen in the rostral preoptic region, followed in decreasing order by the medial preoptic area and the DBB. Nrp1 mRNA was expressed at detectable levels in 38.2±2.5% of GnRH neurons in diestrus (*n* = 4 animals, more than 200 GnRH neurons were considered per animal) and in 50.0±1.5% of GnRH neurons in proestrus (*n* = 4 animals, t_(6)_ = 4.039, *p* = 0.007) with no preferential anatomical distribution. Nrp1 mRNA was also expressed in the ME, the projection field of GnRH neurons (Figure 2B). However, the hybridization signal was not seen in the neural layers, which contain various types of glial cells associated with neuroendocrine axons [14], but instead was detected in the capillary zone (Figure 2B, inset).

To determine whether Nrp1 protein was present in GnRH axon terminals abutting onto the vascular plexus, we performed double immunolabeling studies with antibodies to Nrp1 and GnRH in the ME of the adult brain. Strong Nrp1 immunoreactivity was seen in the external zone of the ME (Figure 2C) at every anteroposterior level examined. Nrp1 labeling was distributed in the same regions as the majority of GnRH axon terminals, and consistent with Nrp1 mRNA expression by GnRH neuronal cell bodies, GnRH-containing fibers were also found to contain Nrp1 protein (Figure 2C, arrows). However, many Nrp1-positive axon-like fibers did not contain GnRH (Figure 2C), suggesting that additional neuroendocrine systems express this receptor. In agreement with *in situ* hybridization data, endothelial cells of the pituitary portal blood vessels were also found to express Nrp1 immunoreactivity (Figure S3).

Thus, the spatial and temporal pattern of expression of Sema3A in the ME together with that of its receptor, Nrp1, in GnRH neurons is consistent with a functional role for Sema3A signaling in promoting GnRH axonal growth towards the vascular plexus at proestrous, when a massive release of the neurohormone into the pituitary portal circulation is required to trigger the preovulatory surge of gonadotropins.

### Sema3A Promotes GnRH Axonal Growth Towards the Endothelial Wall of Portal Blood Vessels

**Figure 3:**
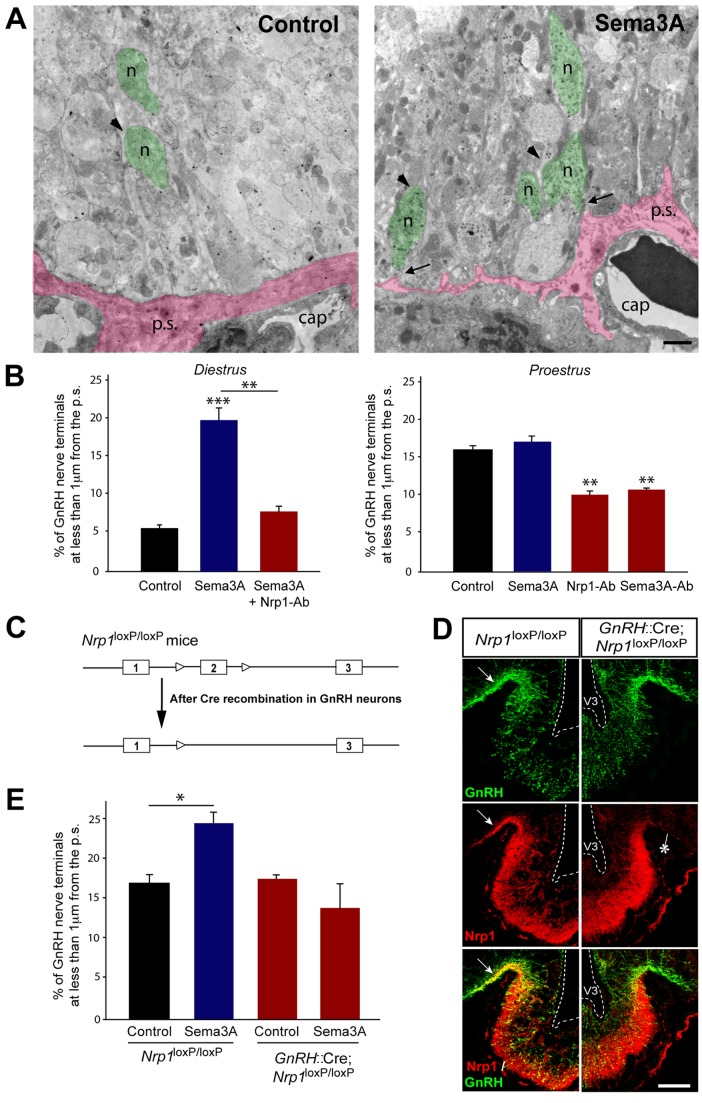
Sema3A-Nrp1 signaling promotes GnRH axonal growth in the ME of the adult female rodent brain. (A) Representative electron micrographs of GnRH-immunoreactive axon terminals (green) from diestrous female rat hypothalamic explants containing the ME, incubated for 30 min in the presence (right panel) or absence (left panel) of Sema3A. Under basal unstimulated conditions (left panel), GnRH nerve endings (n, arrowhead, green) are distant from the pericapillary space (p.s., pink). Sema3A treatment (right panel) causes GnRH axon terminals to advance towards the pericapillary space (p.s., pink), from which they remain separated by only a few nanometers (arrows). Cap, pituitary portal blood capillaries. Scale bar, 1 µm. (B) Quantitative analysis of the percentage of GnRH nerve terminals located less than 1 µm from the pericapillary space in the external zone of the ME, in explants from diestrous (left panel) and proestrous (right panel) rats treated with Sema3A, a Nrp1-neutralizing antibody (Nrp1-Ab), a Sema3A-neutralizing antibody (Sema3A-Ab), both Nrp1-Ab and Sema3A, and in controls. (Left panel) One-way ANOVA, F_(2,11)_ = 54.875, *p*<0.001. (Right panel) One-way ANOVA, F_(3,12)_ = 37.093, *p*<0.001. Tukey's test, ***p<0.001, **p<0.01 for pairs of groups as indicated; *n* = 3–4 animals per group. (C) Genetic strategy to invalidate Nrp1 expression specifically in GnRH-expressing cells in mice. (D) Immunofluorescence analysis of coronal brain sections from adult female *Nrp1* ^loxP/loxP^ (left) and *GnRH::*Cre; *Nrp1* ^loxP/loxP^ (right) littermates using antibodies to GnRH (green) and Nrp1 (red). Note the markedly reduced Nrp1 immunoreactivity in the dorsolateral part of the ME, where most GnRH axon fibers occur, in *GnRH::*Cre; *Nrp1* ^loxP/loxP^ mice (asterisk) when compared to Nrp1^loxP/loxP^ animals (arrow), confirming the efficient ablation of *Nrp1* in GnRH neurons of the former. Scale bar, 50 µm. (E) Quantitative analysis of the percentage of GnRH nerve terminals located less than 1 µm from the pericapillary space in the external zone of the ME in explants from control *Nrp1* ^loxP/loxP^ and *GnRH::*Cre; *Nrp1* ^loxP/loxP^ mice and those treated with Sema3A. One-way ANOVA, F_(3,15)_ = 9.894, *p* = 0.0015. Tukey's test, *p<0.05; *n* = 3–4 animals per group. Data are represented as means ± SEM.

In order to assess whether Sema3A can promote the outgrowth of GnRH axons *in situ*, we analyzed hypothalamic explants containing the ME, maintained *ex vivo* in artificial cerebrospinal fluid. Explants obtained from either diestrous or preovulatory proestrous rats were exposed to 1 µg/ml Sema3A for 30 min, then fixed and processed for electron microscopy. Using 15 nm gold-particle labeling, we revealed a striking transformation of GnRH nerve terminals as a function of the presence or absence of Sema3A in diestrous rats. Indeed, the distance between GnRH nerve terminals (green) and the pericapillary space of pituitary portal blood vessels (p.s., pink) appeared to be significantly shorter in Sema3A-treated explants versus controls (Figure 3A). Quantitative morphometric analysis showed that while the total number of GnRH nerve terminals at a distance of 10 µm or less from the parenchymatous basal lamina (which delineates the pericapillary space) did not vary significantly among treatments (*n* = 4 animals per condition; more than 100 GnRH-immunoreactive axon terminals were considered per explant, one-way ANOVA, F_(2,11)_ = 0.224, *p* = 0.803), their distribution was markedly changed (Figure 3B). In fact, the fraction of GnRH nerve terminals found at a distance of less than 1 µm from the pericapillary space increased by 400% in diestrous ME explants exposed to 1 µg/ml Sema3A for 30 min when compared to controls (Figure 3B, left panel; *n* = 4 hypothalamic explants per condition; *p*<0.001, one-way ANOVA). Importantly, Sema3A-mediated effects on GnRH axonal growth in diestrous explants were abolished upon pretreatment with an Nrp1-neutralizing antibody (Figure 3B, left panel; *n* = 4 hypothalamic explants per condition; *p*<0.01, one-way ANOVA). In contrast, in ME explants obtained from animals in proestrus, when Sema3A is heavily released, GnRH axons naturally extend up to the pericapillary space. In this context, exogenous Sema3A treatment did not further affect the elongation of GnRH nerve terminals towards the pericapillary space (Figure 3B, right panel; *n* = 4 hypothalamic explants per condition; *p*>0.05, one-way ANOVA). However, exposing proestrous ME explants to neutralizing antibodies to either the Nrp1 or Sema3A caused GnRH nerve endings to retract from the pericapillary space (Figure 3B, right panel; *n* = 4 hypothalamic explants per condition; *p*<0.01, one-way ANOVA), suggesting that GnRH axon extension towards the endothelial wall at the transition between diestrus and proestrus is attributable to Nrp1 activation by Sema3A.

### Sema3A-Regulated Structural Changes at the Neurovascular Junction Depend on Nrp1 Expression in GnRH Neurons

To assess whether the structural changes promoted by Sema3A at the GnRH neurovascular junction require neuronal expression of Nrp1, we generated mice in which *Nrp1* expression was selectively knocked out in GnRH neurons. Animals harboring the conditional *Nrp1* allele [29] were crossed with a mouse line expressing Cre recombinase under the control of the endogenous GnRH gene promoter [30] (Figure 3C). To verify the efficacy of our genetic targeting strategy, we analyzed Nrp1 expression in GnRH neurons of wild-type (*Nrp1* ^loxP/loxP^) and mutant (*GnRH::*Cre; *Nrp1* ^loxP/loxP^) littermates by immunofluorescence. In wild-type mice, the expression patterns of GnRH and Nrp1 partially overlapped within the ME (Figure 3D, arrow), as seen in rats (Figure 2C). In contrast, upon Cre-mediated deletion of *Nrp1* in GnRH-positive neurons, Nrp1 expression was abolished in the external zone of the ME where GnRH axons are found (Figure 3D, asterisk), while it was maintained in other neuroendocrine axonal populations (Figure 3D). Electron microscopic analyses of hypothalamic explants from diestrous mice treated with Sema3A (as in Figure 3A,B) confirmed the extension of GnRH nerve terminals towards the pericapillary space in the ME of *Nrp1* ^loxP/loxP^ mice (Figure 3E; *p*<0.05, two-way ANOVA, control versus Sema3A; *n* = 3–4 hypothalamic explants per condition; 150 GnRH-immunoreactive nerve terminals were considered per explant), while this was not observed in *GnRH::*Cre; *Nrp1* ^loxP/loxP^ littermates (Figure 3E; *p* = 0.23, control versus Sema3A), indicating that Nrp1 expression in GnRH neurons is required to mediate this functional regulation by Sema3A *in vivo*.

### Sema3A Promotes Neurite Outgrowth from GnRH Neurons *in Vitro*

**Figure 4:**
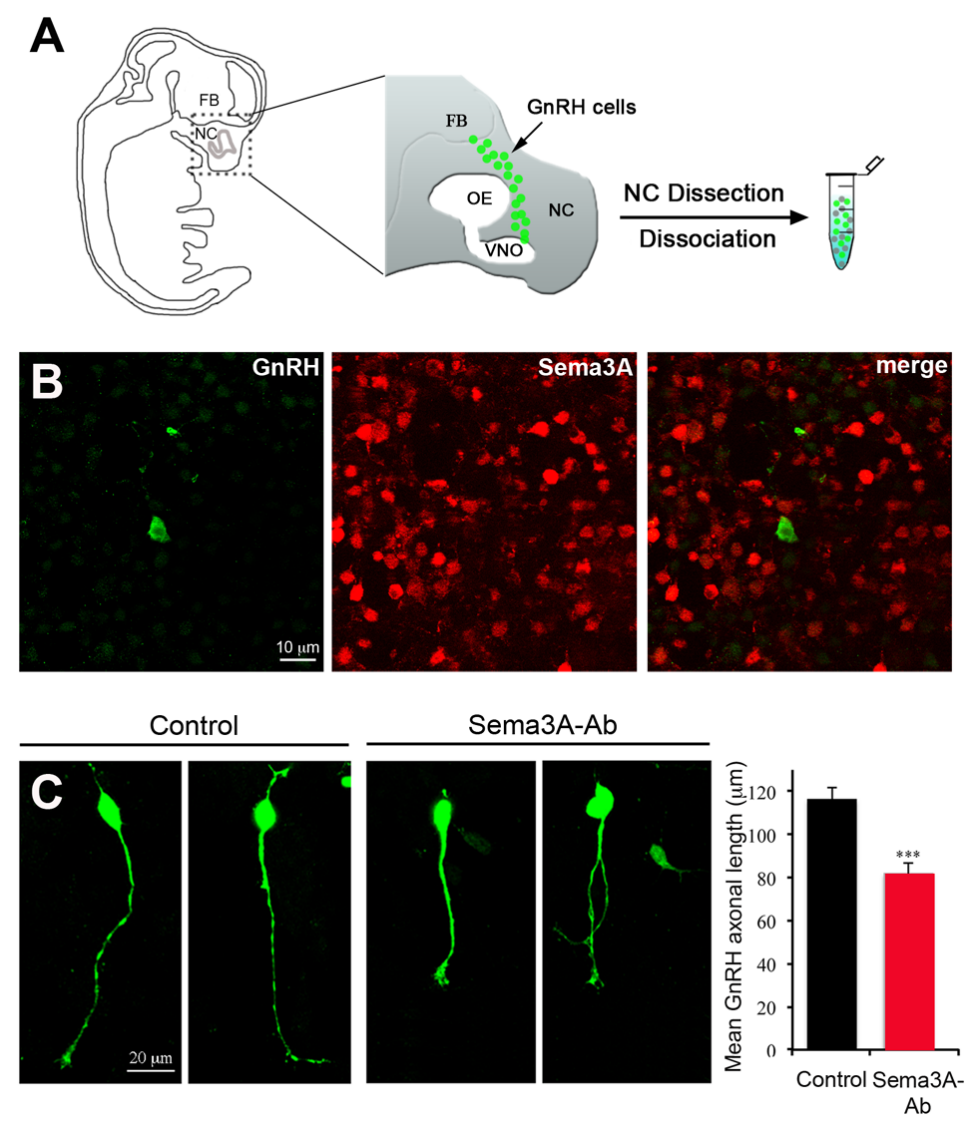
Sema3A immunoneutralization causes the retraction of axon-like processes in primary GnRH neurons *in vitro*. (A) Schematic representation of a sagittal view of a mouse embryo at E12.5, showing the distribution of GnRH neurons (green dots) within the head. Primary cultures were performed from microdissected nasal compartment (NC) explants, which contain most GnRH neurons at this embryonic stage. FB, forebrain; OE, olfactory epithelium; VNO, vomeronasal organ. (B) Representative images showing the binding of the Sema3A-neutralizing antibody (red) to cultured cells surrounding GFP-expressing GnRH neurons (green). (C) Representative images showing the morphology of cultured GnRH neurons under control conditions and after treatment with the Sema3A-neutralizing antibody and bar graph quantifying the mean length of their axon-like processes (control, *N* = 3 independent experiments, *n* = 146 cells; Sema3A-Ab, *N* = 3 independent experiments, *n* = 143 cells). Total number of cultures, 24 from 4 litters. Data are represented as means ± SEM. Unpaired Student's *t* test, *t*_(285)_ = 4.823, *p*<0.0001.

In order to evaluate the role of Sema3A on neurite elongation in GnRH-expressing neurons, we took advantage of our ability to obtain primary cultures of GnRH neurons from the nose of 12.5-d-old *GnRH-GFP* embryos (E12.5) (Figure 4A). As expected, primary GFP-positive neurons were seen to be surrounded by numerous Sema3A-positive cells (Figure 4B), which have been co-isolated with GnRH neurons from the nasal compartment [31]. While 24-h treatment with Sema3A had no effect on GnRH neurite elongation (unpublished data), the addition of a Sema3A-neutralizing antibody to the culture medium for the same time period caused significant shortening of GnRH neuronal processes (Figure 4C). These data strongly suggest that the production of Sema3A by the surrounding cells was responsible for neurite elongation in these GnRH neurons.

**Figure 5:**
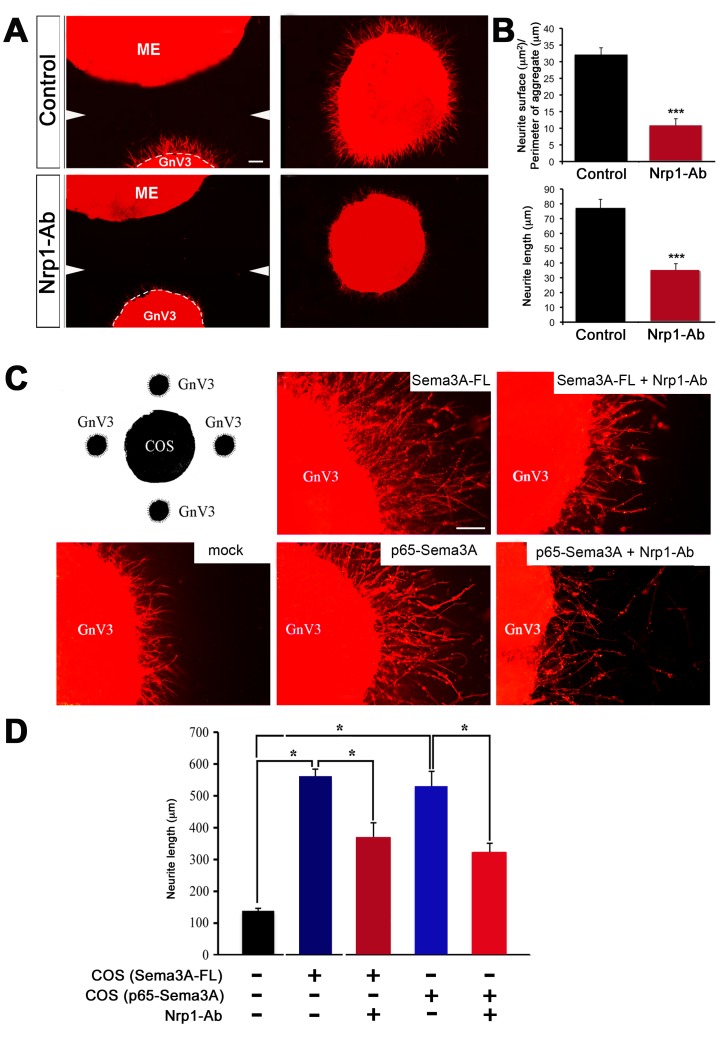
65-Nrp1 signaling is responsible for GnRH neurite sprouting. (A) Three-dimensional matrix assays using co-cultures of ME explants dissected from adult female rats (control, *n* = 3; Nrp1-Ab, *n* = 4) and cell aggregates of immortalized GnV-3 cells, in the absence (top panels) or presence (bottom panels) of an Nrp1-neutralizing antibody (Nrp1-Ab). Co-cultures were fixed and stained with Alexa 588–X phalloidin. GnV-3 cell aggregates show neurite extension under control conditions, whereas neurite sprouting is strongly inhibited by Nrp1-Ab. White arrowheads in the left panels indicate the merging point of the two individual images composing each picture. (B) Quantitative analysis of the area covered by phalloidin staining surrounding the aggregates (top panel; *n* = 3 in controls, *n* = 4 in Nrp1-Ab-treated aggregates; unpaired Student's *t* test, t_(5)_ = 7.424, *p*<0.001) and GnV-3 neurite length (bottom panel; *n* = 3 in controls, *n* = 4 in Nrp1-Ab-treated aggregates; unpaired Student's *t* test, t_(5)_ = 5.610, *p*<0.005), respectively. (C) Co-cultures of GnV-3 cell aggregates placed around aggregates of COS-7 cells transfected with full-length (95 kDa) Sema3A (Sema3A-FL), 65 kDa Sema3A (p65-Sema3A), or the control vector (*n* = 7), in the presence (Sema3A-FL+Nrp1-Ab, *n* = 4; p65-Sema3A+Nrp1-Ab, *n* = 6) or absence of Nrp1-Ab (Sema3A-FL, *n* = 4; p65-Sema3A, *n* = 4), as shown in the schematic drawing. Sema3A-FL and p65-Sema3A are equally effective at inducing GnV-3 neurite growth when compared to control conditions (middle panels), while neurite growth is prevented by the Nrp1-neutralizing antibody (right panels). (D) Quantitative analysis of GnV-3 neurite length (one-way ANOVA with Tukey's *post hoc* test, F_(4,24)_ = 38.058, *p*<0.0001). Data are represented as means ± SEM. Scale bars, 100 µm in (A), 50 µm in (C).

To further explore the role of Sema3A on neurite outgrowth in mature GnRH-expressing neurons, we took advantage of the GnV-3 cell line, one of eleven clones of GnRH-expressing cells obtained by the conditional immortalization of cultured adult rat hypothalamic cells. GnV-3 cells grow in culture in the presence of doxycycline, but stop proliferating and undergo differentiation upon drug removal, exhibiting many of the features of mature adult GnRH neurons, including neurite growth [32]. Rat ME explants were cultured in proximity to aggregates of GnV-3 neuronal cells. After 72 h of co-culture, neurites grew to the same extent on both the proximal and distal sides of GnV-3 cell aggregates (Figure 5A). To test whether Nrp1 was involved in GnRH neurite growth in response to factors released by the ME, Nrp1-neutralizing antibodies were added to the medium. These antibodies significantly attenuated the growth-promoting effect of the ME on GnV-3 neurites (Figure 5B).

Data shown in Figure 1 indicate that endothelial cells of the ME are a major source of p65-Sema3A, the expression of which is induced by estradiol during proestrus. To date, no biological function has been attributed to this 65 kDa isoform of Sema3A. In order to determine whether it is involved in the GnRH axonal-growth-promoting effect described above, we performed a second set of experiments using three-dimensional matrix co-cultures. Briefly, aggregates of GnV-3 cells were cultured for 72 h along with aggregates of mock-transfected COS-7 cells or COS-7 cells secreting the 95 kDa full-length (Sema3A-FL) or recombinant 65 kDa Sema3A, in the presence or absence of the Nrp1-neutralizing antibody (Figure 5C). Remarkably, Sema3A-FL and p65-Sema3A were equally effective at promoting neurite elongation in GnV-3 cells, whereas the Nrp1 antibody stunted this Sema3A-dependent outgrowth (Figure 5D). Altogether these findings suggest that p65-Sema3A, which is highly expressed in the ME during proestrus, unlike the relatively scarce 95 kDa or 125 kDa forms, acts on GnRH neuroendocrine axons through Nrp1 to promote their elongation.

### The Targeted Infusion of Nrp1- or Sema3A-Neutralizing Antibodies into the ME Is Sufficient to Disrupt the Reproductive Cycle

**Figure 6:**
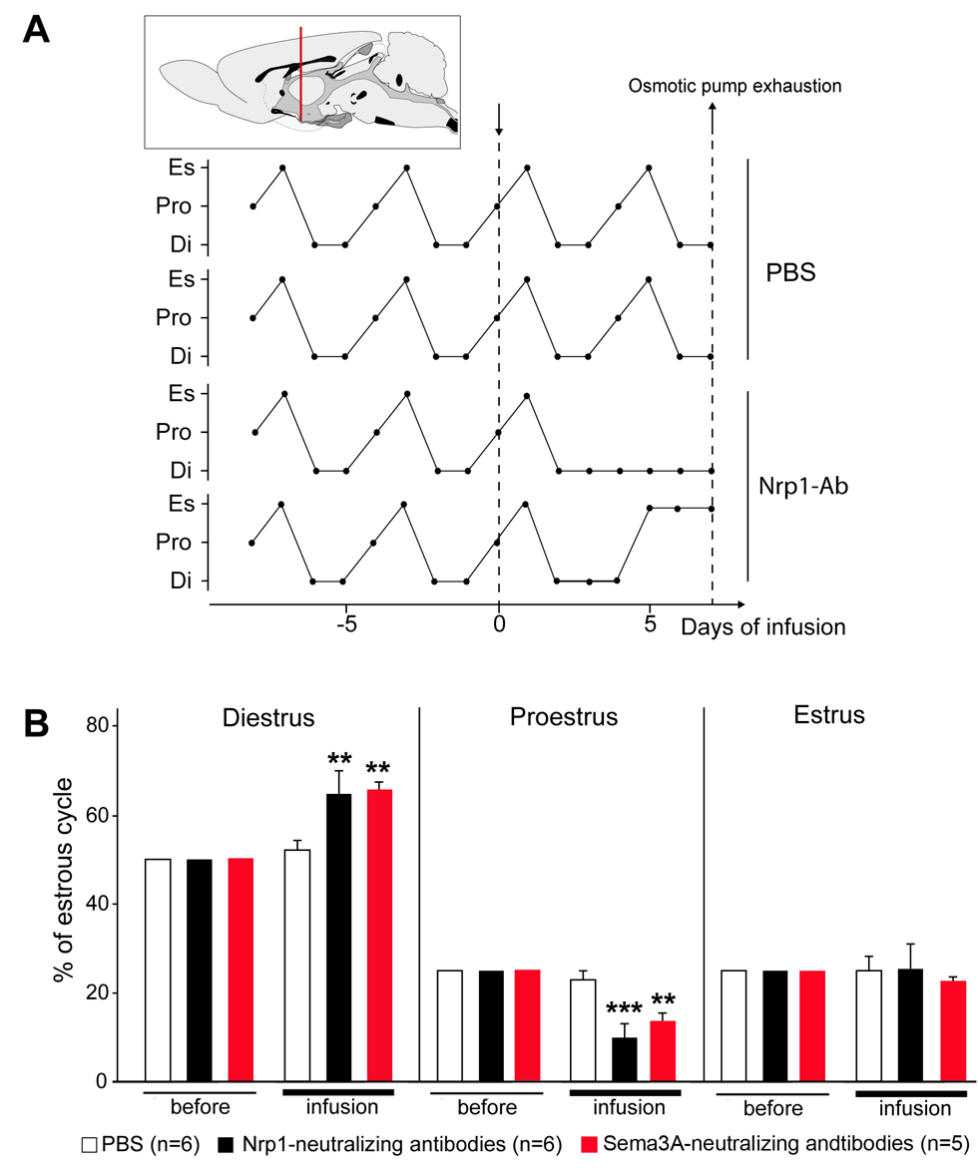
Neutralization of Nrp1 and Sema3A activity in the ME *in vivo* impairs adult reproductive function in rats. (A, Upper panel) Schematic diagram representing the stereotaxic implantation of a 28 gauge infusion cannula connected to a subcutaneously implanted mini-osmotic pump in the ME of cycling female rats, for the delivery of Nrp1- or Sema3A-neutralizing antibodies (0.2 µg/µl, 0.5 µl/h). (Lower panel) Representative estrous cycle profiles showing the disruption of estrous cyclicity by the infusion of Nrp1-Ab but not of PBS into the ME. Infusion was started on day 0 (downward arrow) and ended 7 d later (upward arrow), when the pump contents were exhausted. Di, diestrus; Pro, proestrus; Es, estrus. (B) Quantitative analysis of alterations in ovarian cyclicity (number of days in each phase) caused by Nrp1-Ab or Sema3A-Ab infusion (*n* = 6 animals in the PBS and Nrp1 groups; *n* = 5 animals in the Sema3A group). Diestrus, two-way repeated-measures ANOVA, F_(14,33)_ = 19.073, *p*<0.001, Tukey's test, ** *p* = 0.003 and *p* = 0.005 between before and after infusion within Nrp-1- and Sema3A-treated groups, respectively. Proestrus, two-way repeated measures ANOVA, F_(14,33)_ = 31.119, *p*<0.001, Tukey's test, *** *p*<0.001 and ** *p* = 0.002 between before and after infusion within the Nrp-1- and Sema3A-treated groups, respectively. Estrus, two-way repeated measures ANOVA, F_(14,33)_ = 0.084, *p* = 0.776. Data represented as means ± SEM.

Consistent with the fact that Nrp1 is also expressed in GnRH neurons during embryogenesis [12],[13],[33], we have observed that *GnRH::*Cre; *Nrp1* ^loxP/loxP^ mice exhibit some alterations in the development of the GnRH system, although they display a comparable number of GnRH terminals in the ME as Nrp-expressing mice (Figure 3D,E). To study the physiological relevance of Sema3A-Nrp1 signaling in the mature brain independent of any potential developmental effects, however, we treated adult female rats with a regular 4-d estrous cycle with the Nrp1- or Sema3A-neutralizing antibodies found to inhibit the Sema3A-induced outgrowth of GnRH axon terminals *in situ* (see Figure 3). The antibodies were locally infused into the ME (Figure 6A) at a rate of 0.1 µg/h for 7 d, via a cannula connected to a subcutaneously implanted osmotic minipump. Estrous cycle monitoring by daily inspection of vaginal smears for 1 wk following the initiation of treatment revealed a clear disruption of the cyclic pattern (Figure 6A). In fact, both Nrp1- and Sema3A-antibody-infused animals showed a preponderance of days in the diestrous phase, which is associated with reduced release of GnRH [34] and increased distance of GnRH axon terminals from the pericapillary space [23], and a concomitant reduction of days in proestrus (Figure 6B; *n* = 5–6 per group, *p*<0.05, one-way repeated measures ANOVA, during versus before infusion). In contrast, animals infused with the vehicle alone (PBS) displayed normal 4-d estrous cycles (*n* = 6) (Figure 6A,B). One week after the initiation of treatment, the animals were sacrificed and subjected to control immunoprecipitation or immunofluorescent experiments to verify that the infused Nrp1- and Sema3A- antibodies had successfully targeted receptors and ligands in the ME, respectively (Figure S4 and Figure S5). Immunoprecipitation and immunoblot analyses indicated that the infused Nrp1 antibodies did bind to, and could therefore effectively block, about 50% of the endogenous pool of Nrp1 contained in the ME (Figure S3A). Immunofluorescence analysis of the binding of the Sema3A-neutralizing antibody showed that it selectively targeted the external zone of the ME, where pituitary portal blood vessels and neuroendocrine terminals are localized (Figure S4B). Together with our *ex vivo* results, these data suggest that Sema3A-Nrp1 signaling is required for the neuroendocrine control of the ovarian cycle in the adult rat brain.

### Endothelial-Cell-Derived Sema3A Modulates the Amplitude of the Preovulatory LH Surge

**Figure 7:**
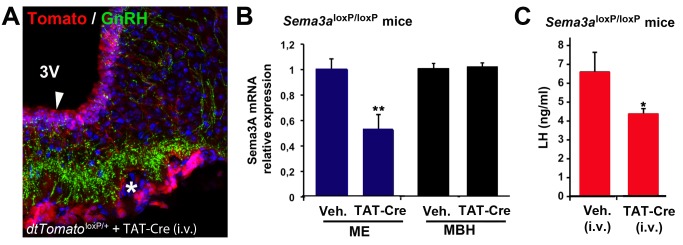
Targeted *Sema3a* gene deletion in endothelial cells of the ME alters the amplitude of the preovulatory GnRH/LH surge in mice. (A) Representative image showing Tomato expression (red) in the capillary zone (asterisk) onto which GnRH axon terminals abut (green) in the ME of *tdTomato* ^loxP/+^ mice into which the TAT-Cre recombinant protein was injected intravenously (i.v.). Note that Tomato is also expressed in tanycytes (arrowhead), whose cell bodies line the floor of the third ventricle (3V) but that do not express Sema3A mRNA (see Figure 1B). (B) Quantitative RT-PCR analysis of Sema3A mRNA expression in the ME, t_(13)_ = 3.372, ** *p* = 0.005, and in the adjacent mediobasal hypothalamus (MBH), t_(10)_ = −0.287, *p* = 0.780, in *Sema3a* ^loxP/loxP^ mice treated i.v. with vehicle (*n* = 7) or TAT-Cre (*n* = 8 and 5, respectively). (C) Preovulatory LH levels in TAT-Cre (*n* = 8) or vehicle-injected (*n* = 7) *Sema3a* ^loxP/loxP^ mice, t_(13)_ = 2.188, *p = 0.048. Data are represented as means ± SEM.

To further study the physiological relevance of Sema3A-Nrp1 endothelial-cell-to-neuron signaling in the mature brain, we used an intravenous injection of the TAT-Cre fusion protein, whose cellular uptake is enhanced compared to Cre recombinase [35] particularly in the ME of living animals [36], to target endothelial cells in *Sema3a* ^loxP/loxP^ mice. Control experiments with *tdTomato* ^loxP/+^ reporter mice showed that a single injection of TAT-Cre into the tail vein caused gene recombination in tanycytes, which do not express Sema3A (see Figure 1C), and in the capillary zone harboring Sema3A mRNA-expressing endothelial cells in adult females (Figure 1B, Figure 7A). Quantitative RT-PCR analyses showed that Sema3A mRNA expression was decreased by 50% in the ME of virgin female *Sema3a* ^loxP/loxP^ mice treated with TAT-Cre and subjected to a male-pheromone-induced preovulatory GnRH/LH surge protocol [37], when compared to vehicle-treated mice (Figure 7C; *n* = 7–8 per group, *t* test, *p*<0.01), while it remained unchanged in the adjacent mediobasal hypothalamus (Figure 7C; *n* = 5–7 per group, *t* test, *p*>0.05), where Sema3A mRNA is abundantly expressed (Figure 1B) and is known to play a key role in the control of GnRH release [38]. This selective attenuation of Sema3A expression in endothelial cells of the ME led to a significant decrease in preovulatory luteinizing hormone (LH) serum levels (Figure 7B; *n* = 7–8 per group, *t* test, *p*<0.05), used as an index of GnRH release [39]. Finally, real-time PCR analyses of Sema3A expression in the ME of wild-type mice across the estrous cycle revealed that Sema3A mRNA levels were significantly higher in proestrus than in diestrus (1±0.11 arbitrary units at proestrus versus 0.54±0.09 arbitrary units at diestrus, *n* = 7 and 3, respectively, t_(8)_ = 2.602, *p* = 0.032). Together, these data suggest that the ovarian cycle modulates Sema3A expression in endothelial cells of the ME, which in turn promotes the elongation of GnRH neuroendocrine axons on the day of proestrus to control the amplitude of the preovulatory GnRH/LH surge.

## Discussion

The reproductive cycle of mammals is critically regulated by hypothalamic GnRH neurons [25], which periodically extend their axons in the ME towards the pericapillary space, into which they release the GnRH neuroendocrine signal during a specific time window [23],[40]. The potential role of vascular endothelial cells in controlling this cyclic growth of axon terminals has not been investigated. Our *in vivo* and *in vitro* findings collectively indicate that Sema3A is a vascular factor promoting GnRH axonal growth in the adult brain and playing a pivotal role in orchestrating the central control of reproduction. We propose that Sema3A released by fenestrated endothelial cells of the hypothalamo-hypophyseal portal blood vessels cyclically induces GnRH neurons to extend their terminals towards the pericapillary space, this directionality being controlled by the glial scaffold along which GnRH axonal fibers travel within the ME (see for review [14]). In turn, this mechanism regulates neuropeptide release at key stages of the ovarian cycle, such as at proestrus, when the preovulatory surge of GnRH occurs.

Our ultrastructural analyses in *GnRH::cre*; *Nrp1* ^loxP/loxP^ mice, which do not exhibit any defect in GnRH axonal targeting when compared to *Nrp1* ^loxP/loxP^ mice (Figure 3D,E), as well as the effect of locally restricted Sema3A infusion on GnRH axonal growth (Figure 3B), strongly indicate that the effects of Sema3A on axonal plasticity within the ME depend on direct Sema3A-Nrp1 signaling in postdevelopmental GnRH terminals. The functional consequence of endothelial Sema3A secretion on GnRH axonal plasticity has, in addition, been demonstrated by the selective invalidation of Sema3A expression in the ME of adult *Sema3a* ^loxP/loxP^ mice by the intravenous injection of the recombinant TAT-Cre protein. Indeed, this approach, which further circumvents any putative developmental effect that might occur with the use of classic promoter-driven Cre expression technology, confirms that the endothelial-Sema3A-promoted elongation of GnRH axons modulates the amplitude of the preovulatory GnRH/LH surge on the day of proestrus.

The molecular pathways that underlie this cyclic Sema3A-Nrp1-mediated GnRH axonal sprouting are unknown, although they appear to be intrinsic to GnRH neurons since Sema3A promotes GnRH neurite outgrowth both in tissue explants and in isolated cell cultures. A recent study has intriguingly suggested that Sema3A could promote axonal growth by inducing protein kinase G activity [41]. Notably, Sema3A receptors are broadly expressed in the axon terminals of other neuroendocrine systems and this signal has been proposed to serve as a coordinator of structural and functional synaptic plasticity in various neuronal circuits [6]. In our study, only about 50% of the Nrp1 expressed in the median emincence was neutralized by antibody infusion. It would be of interest to investigate the effects of more complete Nrp1 invalidation in adult animals, as well as the potential role of endothelial Sema3A in the growth of hypothalamic neuronal projections controlling other anterior pituitary functions, such as the growth-, stress-, and thyroid-hormone axes.

An intriguing finding of this study is that endothelial cells of the ME appear to selectively release the 65 kDa isoform of Sema3A. Interestingly, we show that estradiol mimics ovarian-cycle effects on p65-Sema3A production in ovariectomized rats and that, in agreement with a previous *in vitro* study [42], endothelial cells of the ME express the estrogen receptor ERα. The mechanisms underlying these changes in protein levels within the ME are unknown but likely involve changes in *Sema3a* transcription, rather than its translation or posttranslational processing such as furin cleavage. In line with this idea, analysis of the *Sema3a* gene using the ALGEN PROMO 3.0 software (http://alggen.lsi.upc.es/cgi-bin/promo_v3/promo/promoinit.cgi?dirDB=TF_8.3) predicts the presence of a putative estrogen-receptor-binding element at 100 bp upstream of the transcription initiation site; further experiments will be required to determine whether this presumptive binding site is actually functional. Even though the biological activity of p65-Sema3A has been validated in heterologous systems mimicking growth-cone collapse [43] and in co-culture systems using sympathetic ganglion explants [22], this isoform was originally described as a proteolytic by-product of p95-Sema3A, with reduced functional activity [22]. Similarly, it has been reported recently that the anti-angiogenic activity of the 61 kDa proteolytic fragment of Sema3B is dramatically reduced compared to the full-length 83 kDa isoform [44]. In contrast, here we demonstrate that 65 kDa and 95 kDa Sema3A isoforms are equally effective at promoting GnRH neurite elongation ex vivo (Figure 5C,D), indicating that the proteolytic cleavage of Sema3A does not interfere with its axonal-growth-promoting activity. In conjunction with the fact that the expression of the 65 kDa isoform of Sema3A, unlike the 125 kDa precursor and the best known 95 kDa secreted isoform, is subject to cyclic changes, being maximal on the day of proestrus, these results uncover for the first time a physiological role for p65-Sema3A in the adult brain.

In conclusion, we show that in the ME of the hypothalamus, p65-Sema3A is an endothelial-cell-derived protein that acts on Nrp1 receptors in GnRH neuroendocrine processes, which have previously been seen to express axonal markers such as GAP-43 [27], to promote their growth towards the target vascular wall during a time window of the reproductive cycle that is critical to ovulation. Because ovarian-cycle-regulated GnRH axonal elongation in the adult brain is likely to depend on the coordinated action of many extracellular factors, endothelial p65-Sema3A may work in concert or in competition with other secreted molecules including VEGF, nitric oxide, TGF-β1, and BDNF, which are particularly enriched in the capillary zone of the ME [21],[36],[45],[46] and may influence axonal plasticity by modulating the endothelial expression of or responsiveness to semaphorins [8],[47]–[49]. These findings have implications for the possible roles of p65-Sema3A in adult brain function. Finally, our results raise the intriguing possibility that vascular semaphorins may play important and unexpected roles in the adult neural plasticity underlying several other key physiological processes such as learning, stress, and the control of energy homeostasis [50]–[53].

## Methods

### Ethics Statement

All experiments were performed in accordance with the European Communities Council Directive of November 24, 1986 (86/609/EEC) regarding mammalian research and were approved by the Institutional Animal Care and Use Committee of Lille and the animal experimentation committee of the Royal Netherlands Academy of Arts and Sciences in Amsterdam.

### Animals

#### Rats

Female Sprague Dawley rats (Janvier, Saint-Berthevin, France) weighing 250–300 g were used for *in situ* hybridization, immunohistofluorescence, immunoprecipitation, Western blotting of tissue explants, electron microscopy, and intracerebral infusion experiments. Vaginal smears were examined daily, and only rats that exhibited at least two consecutive 4-d estrous cycles were used for experiments. Diestrus 1 and 2 were defined by the predominance of leukocytes in the vaginal lavage, the day of proestrus was characterized by the predominance of round nucleated epithelial cells, and estrus was distinguished by a large number of clustered cornified squamous epithelial cells.

#### C57BL/6 mice

*Nrp1* ^loxP/loxP^ [29] and *tdTomato* ^loxP/+^ mice were purchased from JAX mice (Jackson laboratory, Maine, USA), while *Sema3a* ^loxP/loxP^ mice were generated as described previously [54]. *GnRH::Cre* ^+/−^ mice were a generous gift from Dr. Catherine Dulac (Howard Hughes Medical Institute, MA) [30]. *GnRH-GFP* mice were kindly provided by Dr. Daniel J. Spergel (Section of Endocrinology, Department of Medicine, University of Chicago, IL) [55]. Animals were genotyped by PCR using tail DNA samples. Genomic DNA was extracted using the NucleoSpin Tissue kit (Cat. No. 740.952.250, Macherey-Nagel, Hoerdt, France). PCR was carried out using GoTaq DNA Polymerase (Promega, USA) under the following cycling conditions: 95°C, 2 min; 95°C, 1 min; 57.3°C, 1 min; 72°C, 1 min, 35 cycles; 72°C, 5 min; 4°C until analysis. The primer sequences used were Cre-sense 5′-ATGGCTAATCGCCATCTTCC-3′, Cre-antisense 5′-CTGGTGTAGCTGATGATCCG-3′; Nrp1-sense 5′-AGGTTAGGCTTCAGGCCAAT-3′, Nrp1-antisense 5′-GGTACCCTGGGTTTTCGATT-3′; Tomato-sense 5′-CTGTTCCTGTACGGCATGG-3′, Tomato-antisense 5′-GGCATTAAAGCAGCGTATCC-3′.

Adult (2–4-mo-old) female rats and mice were housed in a room with controlled photoperiod (12 h/12 h light cycle) and temperature (21–23°C). Animals were allowed access to tap water and pelleted food *ad libitum*.

### Primary Culture of ME Endothelial Cells

The purification of endothelial cells of the ME was realized by sequential immunopanning. Endothelial cells of the ME were isolated from 10-d-old rats using a procedure adapted from a protocol kindly provided by Dr. Ben Barres (Stanford, CA) [20], as described previously [21]. In brief, ME explants were enzymatically dissociated at 37°C for 90 min using a solution of papain (33 U/ml) (Worthington/Cooper, Lakewood, NJ) in MEM/HEPES (Invitrogen) containing L-cysteine (0.4 mg/ml) (Sigma) and DNase (125 U/ml) (Sigma). Tissues were then triturated in a solution containing ovomucoid trypsin inhibitor solution (2 mg/ml) (Boehringer Mannheim, Mannheim, Germany), DNase (125 U/ml), and BSA (1 mg/ml) (Sigma), to obtain a suspension of single cells. The suspension was filtered through a 20 µm nylon mesh. After centrifugation at 550×*g*, single cells were successively panned on a Petri dish coated with an anti-CD90 mouse monoclonal antibody, which recognizes the rat Thy1.1 antigenic determinant (MRC-OX7; Serotec, Oxford, UK) to deplete macrophages and fibroblasts, and on a second Petri dish coated with rat neural antigen (RAN)-2 ascites (LGC Promochem, Molsheim, France) to deplete meningeal cells and type-1 astrocytes; the remaining cells were incubated in a Petri dish coated with an affinity-purified rabbit antibody raised against PV1, which selectively recognizes fenestrated vascular endothelial cells of the ME [18]. Purified endothelial cells were cultured in DMEM supplemented with 10% fetal bovine serum, 1% L-glutamine, and 2% penicillin/streptomycin until they reached confluency. They were then recovered by trypsin digestion and plated in 10 cm dishes or on poly-D-lysine (Sigma) coated coverslips. Three primary cultures from three independent litters were used in the present study.

To produce endothelial cell-conditioned medium (EC-CM), cell monolayers were cultured in 10-cm dishes for 48 h in DMEM (devoid of phenol red; Invitrogen) supplemented with 1% L-glutamine, 1% penicillin/streptomycin, 5 µg/ml insulin (Sigma), and 100 µM putrescine (Sigma). For Western blot analysis, 10 ml of EC-CM were concentrated using a Centriplus centrifugal filter device (size cutoff of 10 kDa, Cat. 4411, YM10; Millipore, Bedford, MA) to obtain a final volume of 30–40 µl. The concentrated medium was mixed with NuPAGE LDS sample buffer 4× (Invitrogen) to obtain a final concentration of 1×, boiled for 5 min, and stored at −80°C until loading.

### Inhibition of Furin Proteolytic Activity in Mouse Endothelial Cells

Confluent cultures of mouse endothelial cells SVEC4-10 were incubated in serum-free DMEM medium for 48 h, either in presence or absence of the furin protease selective inhibitor Dec-RVKR-CMK (100 µM; Bachem). Cell-conditioned media were concentrated with a size cutoff of 50 kDa (Vivaspin, Sartorius), and a sample size equivalent to the medium collected from a 2 cm^2^ cell monolayer was separated by SDS-PAGE and eventually analyzed by Western blotting.

### Reverse Transcription-PCR Amplification

Complementary DNA fragments derived from mRNAs encoding Sema3A were generated by reverse transcription (RT)-PCR of total RNA extracted from the neonatal rat brain, adult female rat ME, or primary cultures of ME endothelial cells. One µg of Trizol (Life Technologies, Grand Island, NY)-extracted RNA was reverse transcribed to cDNA in a final volume of 10 µl containing 200 U of SuperScript II reverse transcriptase (Invitrogen), 20 U of RNase inhibitor (Promega, Madison, WI), and 0.5 µg of oligo-dT primer. After a 1 h incubation at 42°C, the reaction was stopped by heating at 94°C for 5 min. PCR was performed by using 1 µl of each reverse transcription reaction and Hotstart Taq DNA polymerase (Qiagen, France) in a volume of 50 µl. The thermocycling conditions were 15 min at 95°C for enzyme activation, followed by 35 cycles at 94°C for 1 min, annealing at 53°C for 1 min, 72°C for 1 min, followed by a final extension period of 10 min at 72°C. A 364 bp DNA fragment (sense 5′-TCATCCTGAGGACAACAT-3′, antisense 5′-GCATATCTGACCTATTCT-3′) corresponding to nucleotides 444–807 (NM017310) was amplified. PCR with the substitution of cDNA with RNA served as a control. All cDNAs generated by RT-PCR were verified by sequencing. β-actin cDNA was amplified with primers 5′-AACTGACAGACTACCTCA-3′ and 5′-GCTCATAGCTCTTCTCCA-3′ to verify the quality of samples (not shown).

### In Situ Hybridization

#### Tissue preparation

The brains of adult female rats (*n* = 4 per experiment) were fixed by transcardiac perfusion with ice-cold 4% paraformaldehyde in 0.1 M borate buffer at pH 9.5, postfixed in the same fixative containing 10% sucrose for 2 h at 4°C, and immersed in 20% sucrose in 0.02 M potassium phosphate buffered saline prepared with DEPC-treated water at 4°C overnight, embedded in Tissue-Tek (Miles, Elkhart, IN), and frozen in liquid nitrogen. Coronal sections (30 µm) cut on a cryostat were mounted onto gelatin-subbed and poly-L-lysine coated slides, dried under vacuum for 4 h, and stored in boxes with dessicants at −80°C until use.

#### ^33^P-labeled cRNA probes

Plasmids were provided by Dr. Marc Tessier-Lavigne (University of California, San Francisco). The lyophilized plasmid vector pBluescript II SK containing a PstI/BamHI fragment of 1,181 bp corresponding to nucleotides 429–1610 (Genbank X85993) of the mouse Sema3A complementary DNA (cDNA) was used. To generate antisense ^33^P-labeled cRNA, the plasmids were linearized by digestion with NotI and subjected to *in vitro* transcription with T7 RNA polymerase. For generation of sense ^33^P-labeled cRNA, the plasmids were linearized by digestion with XhoI and subjected to *in vitro* transcription with T3 RNA polymerase, according to previously described protocols [45]. The lyophilized plasmid vector pBluescript II SK containing a 1,285 bp PstI fragment of rat Nrp1 corresponding to 490 bp of the 5′UTR and 795 bp of the coding region was used. SacI and T3 RNA polymerase were used to synthesize the antisense probe, and HindIII and T7 RNA polymerase were used to synthesize the sense probe.

#### Digoxigenin-labeled GnRH cRNA probe

The plasmid vector GST7 containing a 330 bp BamHI/HindIII insert of GnRH cDNA was linearized with HindIII for antisense and with BamHIII for sense probes. The riboprobes were synthesized *in vitro* in a 10 µl transcription reaction volume containing 1 µg of linearized GnRH cDNA, 1 µl of a 2 mM solution of Digoxigenin-11-dUTP (Roche Diagnostics, Meylan, France), 2 µl of a mixture of GTP, CTP, and ATP (2.5 mM each) diluted from 10 mM stocks, 1 µl of DTT 100 mM, 1 µl of RNasin, RNA polymerase (T7 for antisense and SP6 for sense), and 10× transcription buffer. This mixture was incubated at 37°C (T7) or at 40°C (SP6) for 1 h. Residual DNA was digested with DNAse.

#### In situ hybridization

Single-label *in situ* hybridization for Sema3A or for Nrp1 was performed as we have previously described [45]. Briefly, after proteinase K digestion (10 µg/ml at 37°C; Boehringer Mannheim, Indianapolis, IN) and acetylation (0.0025% acetic acid at room temperature) for 30 min, the sections were dehydrated through an ascending ethanol series and dried under vacuum for 4 h. The ^33^P-labeled cRNA probes were heated at 65°C for 5 min with 500 µg/ml yeast tRNA (Sigma, Saint Quentin Fallavier, France) and 50 µM dithiothreitol (DTT) (Euromedex) in DEPC (Sigma)-treated water and then diluted to an activity of 5×10^6^ cpm/µl with hybridization buffer containing 50% formamide (Boehringer Mannheim), 0.25 M sodium chloride, 1× Denhardt's solution (Sigma), and 10% dextran sulfate (Pharmacia). Eighty µl of this hybridization solution were pipetted onto the sections, which were covered with a glass coverslip and sealed with DPX (Electron Microscopy Sciences) before incubation for 20 h at 58°C. Then, the slides were washed four times (5 min each) in 4× Saline Sodium Citrate (SSC) before digestion with RNase (20 µg/ml for 30 min at 37°C; Sigma) and rinsed at room temperature in decreasing concentrations of SSC (2×, 1×, 0.5× for 10 min each) containing 1 mM DTT, to final stringency in 0.1× SSC at 65°C for 30 min. After dehydration in an ascending ethanol series, the sections were vacuum-dried, dipped in NTB-2 liquid emulsion (Kodak), dried, and stored in the dark. Emulsion-coated slides were developed after 1 mo for Sema3A or after 14 d for Nrp1 with Kodak D-19 developer. The sections were then counterstained with thionine, dehydrated, cleared in xylenes, and mounted with DPX.

For double-label *in situ* hybridization, prehybridization, hybridization, and posthybridization procedures were similar to those described above, with the exception that the sections were not dehydrated after the last 0.1× SSC rinse, but were further processed for the localization of digoxigenin-labeled hybrids. Briefly, after rinsing in 2× SSC, sections were blocked overnight with 2× SSC buffer containing 0.3% Triton X-100 and 2% normal goat serum, then washed in buffer 1 (100 mM Tris-HCl and 150 mM NaCl, pH 7.4), and incubated for 5 h with alkaline-phosphatase-conjugated anti-digoxigenin (Roche) diluted 1∶1,000 with buffer 1 containing 1% normal goat serum. After rinsing in buffer 1 and in buffer 2 (100 mM Tris-HCl, 50 mM MgCl2, and 100 mM NaCl, pH 9.5), sections were incubated in nitro blue tetrazolium (NBT)+5-bromo-4-chloro-3-indolyl phosphate (BCIP) chromogen solution (Sigma) for 12 h, and the reaction stopped with buffer 3 (10 mM Tris-HCl, 1 mM EDTA, pH 8). Sections were then quickly dehydrated in ethanol, dried, and dipped in K5 emulsion (Ilford, Saint-Priest, France).

#### Quantitative analysis

Nrp1 mRNA was quantified in GnRH neurons as follows. GnRH mRNA-expressing cells were observed under brightfield illumination, and Nrp1 mRNA-expressing cells were observed under darkfield illumination. Thus, each digoxigenin-labeled GnRH neuron was examined for the presence of silver grains, indicating Nrp1, using alternate bright- and darkfield observations. About 200 GnRH cells were studied per animal. The total number of GnRH mRNA-expressing cells and the number of GnRH cells coexpressing Nrp1 mRNA were counted per section. From these data, the proportion of GnRH neurons expressing Nrp1 mRNA per animal was calculated and averaged.

### Fluorescence-Activated Cell-Sorting Analysis

MEs from female P90 rats (*n* = 3) were microdissected and enzymatically dissociated using a Papain Dissociation System (Worthington, Lakewood, NJ) to obtain single-cell suspensions. Subsequently, dissociated cells were resuspended in Hanks' balanced salt solution (HBSS; Invitrogen) containing 1% BSA. Endothelial cells were labeled for 30 min at 4°C using an affinity-purified rabbit antibody raised against PV1 (1∶100), which selectively recognizes fenestrated vascular endothelial cells of the ME [18], followed by a 15-min incubation at 37°C with an AlexaFluor 488 anti-rabbit secondary antibody (1∶100, Invitrogen).

FACS was performed using an EPICS ALTRA Cytometer device (Beckman Coulter, Inc.). Sorted GFP-positive cells and GFP-negative cells (yield: 30,000 cells isolated from each animal) were collected into two separate tubes containing 500 µl of sterile HBSS (Invitrogen) and subsequently centrifuged for 1 min at 7,500 *g* (maximum) to pellet the cells. HBSS was then aspired and 8 µl of a solution containing 1 µl of 0.1% Triton X-100 and 7 µl of Prime RNase inhibitor (diluted 1∶100 in diethylpyrocarbonate-treated water; Invitrogen) was added. Captured cells were used to synthesize first-strand cDNA using the SuperScript III First-Strand Synthesis System for RT-PCR (Invitrogen) following the manufacturer's instructions. Controls without reverse transcriptase were performed to demonstrate the absence of contaminating genomic DNA. RNA isolated from the adult rat brain was also reverse transcribed and used as a positive control. PCR was performed at 35 cycles on a thermocycler (30 s denaturation at 94°C, 30 s annealing at 55–65°C, and 2 min elongation at 72°C). PCR primer pairs were as follows: Sema3A forward primer, 5′-ATGAATGCAAGTGGGCTGGA-3′; Sema3A reverse primer, 5′-CGGTCCTGATGGGATGATGG-3′; PV1 forward primer, 5′- TGAAGGAGGGCAACAAGACC-3′ PV1 reverse primer, 5′-AACGGTAGACCAGCGAATCC-3′; β3-tubulin forward primer, 5′-CGTCTCTAGCCGAGTGAAGTC-3′; β3-tubulin reverse primer, 5′-TCCGAGTCCCCCACATAGTT-3′; DARP32 forward primer, 5′-CCTCATAGAGCGCGGGATTT-3′; DARP32 reverse primer, 5′-CGGATCATCTCCACCTGTCG-3′; TSH forward primer, 5′-GAGAGTGTGCCTACTGCCTG-3′; TSH reverse primer, 5′-CATCCCGGTATTTCCACCGT-3′; GAPDH forward primer, 5′-GGACCAGGTTGTCTCCTGTG-3′; GAPDH reverse primer, 5′-ATTCGAGAGAAGGGAGGGCT-3′. Qualitative RT-PCR experiments were run three times on sorted cells from three different animals. Real-time PCR was carried out on Applied Biosystems 7900HT Fast Real-Time PCR System using exon-boundary-specific TaqMan Gene Expression Assays (Applied Biosystems): PV1 (PV1_Rn00571706_m1), Sema3A (Sema3A_Rn00436469_m1), ERα (Esr1_Rn01640372_m1), ERβ (Esr2_Rn00562610_m1), and housekeeping ribosomal RNA (Rn45_Rn03928990_g1).

### Immunohistochemistry

Two-month-old female rats and monogenic and bigenic mouse littermates were perfused transcardially with 4% paraformaldehyde in 0.1 M PBS, pH 7.4. Rat and mouse brains were postfixed in the same fixative containing 20% sucrose for 2 h at 4°C, immersed in 20% sucrose in 0.1 M phosphate buffered saline overnight at 4°C, embedded in Tissue-Tek, and frozen in liquid nitrogen. Coronal sections (14 µm) were cut on a cryostat and mounted onto chrome-alum-gelatin–coated slides and subjected to fluorescent labeling. Briefly, the sections were washed in 0.1 M PBS, then incubated for 10 min at room temperature in blocking solution containing 2% normal donkey serum (D9663; Sigma) and 0.3% Triton X-100 in 0.1 M PBS. Sections were then incubated overnight at 4°C with the primary antibodies diluted in the same solution. Primary antibodies used were rabbit polyclonal antibodies diluted 1∶3,000 for GnRH [56] and PV-1 [18] and a goat polyclonal antibody to the extracellular domain of rat Nrp1 (AF 566; R&D Systems) diluted 1∶400. Sections were washed in 0.1 M PBS, and labeling revealed by incubation for 1 h at room temperature with AlexaFluor 568–conjugated anti-rabbit or AlexaFluor 488–conjugated anti-rabbit antibodies (1∶400; Molecular Probes) or biotin-conjugated donkey anti-goat IgGs (1∶400; Jackson Immunoresearch, West Grove, PA) followed by AlexaFluor 488– or AlexaFluor 568–conjugated streptavidin (1∶500) for 1 h. Vascular endothelial cells were visualized with tetramethylrhodamine isothiocyanate (TRITC)-conjugated *Bandeiraea simplicifolia* lectin (1∶600; Sigma). After washes, slices were coverslipped with Permafluor medium (434990; Immunon, Pittsburgh, PA).

For the detection of Sema3A, female mouse brains were quickly harvested, embedded in ice-cold Tissue Tek, frozen in isopentane (−55°C), and stored at −80°C until use. Brains were cut into 20-µm-thick coronal sections and processed for immunohistochemistry as follows. Slide-mounted sections were (1) fixed by immersion for 1 min in methanol/acetone (vol/vol) at −20°C; (2) blocked for 30 min using a solution containing 4% normal goat serum and 0.3% Triton X-100; (3) incubated overnight at 4°C with a rabbit polyclonal anti-Sema3A (1∶50, sc-10720; Santa Cruz Biotechnology, Santa Cruz, CA), which selectively recognizes Sema3A in Western blots (Figure 1E,F,G and Figure S4), and a rat anti-mouse PV1 (MECA32 clone, 1∶200; gift from Professor Britta Engelhardt, Switzerland) followed by 1 h at room temperature with a cocktail of secondary AlexaFluor-conjugated antibodies (1∶500, Molecular Probes, Invitrogen, San Diego, CA); and (4) counterstained with Hoechst (1∶10,000, Molecular Probes, Invitrogen) and coverslipped using Mowiol (Calbiochem, USA).

### Protein Extraction and Immunoprecipitation from Tissue Explants

MEs were obtained from cycling diestrous and proestrous rats killed at 16 h. After dissection, each fragment was placed in a microcentrifuge tube, snap frozen in dry ice, and stored at −80°C. Protein extracts of a set of two MEs were prepared by trituration of the fragments through 22 and 26 gauge needles in succession in 200 µl of lysis buffer (25 mM Tris, pH 7.4, β-glycerophosphate, 1.5 mM EGTA, 0.5 mM EDTA, 1 mM sodium pyrophosphate, 1 mM sodium orthovanadate, 10 µg/ml leupeptin and pepstatin A, 10 µg/ml apoprotinin, 100 µg/ml PMSF, and 1% Triton X-100) for straight analysis, or in 750 µl for immunoprecipitation. After 30 min of gentle rocking at 4°C, the tissue lysates were cleared by centrifugation at 14,000 rpm for 15 min. For straight analysis, the protein content of supernatants was determined using BCA protein assays (Pierce Chemical, Rockford, IL), and equal amounts of proteins were mixed with SB4X to obtain a final volume of 50 microliters in 1× NuPAGE LDS sample buffer (Invitrogen). For immunoprecipitation, 60 µl of protein A-sepharose (1∶1 slurry in lysis buffer, P3391; Sigma) were added to the supernatants in order to remove endogenous IgGs (preclearing). The samples were then rocked for 30 min at 4°C, the beads were centrifuged for 15 s at 14,000 rpm, and the supernatants collected. Equal amounts of protein (350 µg) in 750 µl of lysis buffer were incubated with 2 µg of anti-Nrp1 (AF566, R&D Systems) with gentle rocking overnight at 4°C. Thereafter, 60 µl of protein A-sepharose beads were added to the antibody-antigen complex and incubated for 3 h at 4°C. The sepharose beads were collected by centrifugation. Beads were then washed twice with ice-cold lysis buffer, and boiled for 5 min in 50 µl of 2× NuPAGE LDS sample buffer (Invitrogen). Samples were stored at −80°C until use.

### Western Blotting

Samples were boiled again for 5 min after thawing and electrophoresed for 1 h at 150 V in precast 3%–8% Tris-acetate gels or for 35 min at 200 V in precast 4%–12% MES polyacrylamide-SDS gels (Invitrogen). Then, the proteins were transferred onto 0.2 µm pore-size polyvinylidene difluoride (PVDF) membranes (Invitrogen) for 1 h at room temperature (RT). Blots were incubated for 1 h in Tris-buffered saline (TBS; 0.05 M Tris, pH 7.4, 0.15 M NaCl) with 0.05% Tween 20 (TBS-T) and 5% nonfat milk at RT, or in TBS with 1% Tween 20 for 1 h at RT. The membranes were exposed to the primary antibody (goat polyclonal anti-Nrp1, 1∶100, AF566, R&D Systems, or rabbit polyclonal anti-Sema3A, 1∶100, sc-10720, Santa Cruz Biotechnology) diluted in TBS-T with 5% nonfat milk overnight at 4°C with gentle rocking. Immunoreactions were detected with horseradish peroxidase-conjugated secondary antibodies (Sigma) in TBS-T with 5% nonfat milk for 1 h at room temperature, and developed using enhanced chemiluminescence (NEL101; PerkinElmer, Boston, MA). When necessary, the membranes were stripped (PBS; 5 min at 100°C) and incubated with a goat polyclonal antibody against actin (1∶1,000; Santa Cruz Biotechnology). Protein expression was densitometrically analyzed using Scion Image software (Scion Corporation, MA).

### Assessment of Ultrastructural Changes in GnRH Nerve Terminals Induced by Sema3A

To determine whether Sema3A promotes GnRH nerve terminal plasticity, *ex vivo* experiments were carried out according to previously described protocols [21]. Female rats weighing 250–300 g were killed on diestrus 2 (*n* = 12) or proestrus (*n* = 12) by decapitation. Four animals were used per condition. After rapid removal of the brain, hypothalamic explants were microdissected without damaging the MEs. Explants were placed in 12-well plates and preincubated for 30 min at 37°C in 1 mL of Krebs-Ringer bicarbonate buffer, pH 7.4, containing 4.5 mg/ml D-dextrose and 5 µM tetrodotoxin, with or without Nrp1- or Sema3A-neutralizing antibodies (15 µg/ml, AF566 and MAB-1250, respectively, R&D Systems), under an atmosphere of air containing 5% CO_2_. The Nrp1-neutralizing antibody has been shown to selectively target the semaphorin-binding domain of Nrp1 [13]. In addition, we confirmed the specificity of the Sema3A-neutralizing antibody, which specifically detects Sema3A in the conditioned media from transfected COS-7 cells (Figure S4C), using immunohistochemistry (Figure S4B). After this preincubation, tissues were placed in fresh medium with or without a recombinant human Semaphorin-3A/Fc chimera (1,000 ng/ml; 1250-S3, R&D Systems) for an additional 30-min incubation period. Explants were then processed for electron microscopy as described previously [23]. Briefly, tissues were fixed by immersion in a solution of 2% paraformaldehyde, 0.2% picric acid, and 0.1% glutaraldehyde in 0.1 M phosphate buffer, pH 7.4, for 2 h at 4°C. Tissues were postfixed with 1% OsO_4_ in phosphate buffer for 1 h at room temperature. After dehydration, tissues were embedded in Araldite. Semithin sections (1–2 µm thick) were used to progressively approach and identify the portion of the ME targeted for ultrastructural studies—that is, the area where the pituitary stalk becomes distinct from the base of the hypothalamus but still remains attached to it by the hypophyseal portal vasculature [23]. This area, which does not extend beyond 20 µm, contains high numbers of GnRH fibers. To detect GnRH immunoreactivity, ultrathin sections (80–90 nm thick) collected on Parlodion 0.8%/isoamyl acetate-coated 100 mesh grids (EMS, Fort Washington, PA) were treated using an immunogold procedure described previously [23]. Briefly, after a preliminary treatment with H_2_O_2_ (10%; 8 min) and a blocking step in TBS (0.1 M Tris, pH 7.4, 0.15 M NaCl) containing 1% normal goat serum and 1% bovine albumin serum (TBSB) (10 min at room temperature), the grids were floated on a drop of the following reagents and washing solutions: (1) rabbit anti-GnRH (1∶5,000) in TBSB for 60 h at 4°C, (2) TBS to remove excess antibodies (three times for 10 min), (3) colloidal gold (18 nm)-labeled goat anti-rabbit immunoglobulins (Jackson ImmunoResearch) 1∶20 in TBS for 90 min at room temperature, (4) TBS (three times for 10 min), and (5) distilled water (three times for 10 min). The sections were then counterstained with uranyl acetate and lead citrate before observation. The specificity of the GnRH antisera used has been discussed previously [56]. Ultrathin immunolabeled sections were examined with a Zeiss transmission electron microscope 902 (Leo, Rueil-Malmaison, France), and images were acquired using a Gatan Orius SC1000 CCD camera (Gatan France, Grandchamp, France). Morphometric analysis was performed by an investigator blind to hypothalamic explant treatment on digitalized images taken at an original magnification of 12,000× from 10–15 ultrathin sections per animal, with a space of 25 sections between them, to avoid taking the same GnRH nerve terminal into consideration twice (the diameter of a GnRH nerve terminal rarely exceeds 2 µm). All GnRH-immunoreactive nerve terminals located at less than 10 µm from the parenchymatous basal lamina (i.e., the pial surface of the brain) were taken into consideration—that is, more than 100 distinct axon terminals per animals (i.e., almost all GnRH nerve terminals abutting onto the pituitary portal blood vessels in the aforementioned 20-µm-thick region of the ME). Immunolabeled terminals confined to a distance of 10 µm or less from the basal lamina were imaged and the distance from the nerve terminal to the pericapillary space recorded.

Similar electron microscopic analyses were performed in 60-d-old diestrous *Nrp1* ^loxP/loxP^ (*n* = 8) and *GnRH*::Cre; *Nrp1* ^loxP/loxP^ (*n* = 8) mice. Three to four animals were used per condition.

### Functional Assay in GnRH Primary Cultures

Timed-pregnant *GnRH-GFP* mice were anesthetized with an intraperitoneal injection of 200 mg/kg ketamine and killed by cervical dislocation. E12.5 embryos were harvested, and nasal regions were dissected from each embryo and dissociated using the Papain Dissociation System (Worthington, Lakewood, NJ) to obtain single-cell suspensions.

Dissociated nasal tissue containing *GnRH-GFP* cells, mesenchymal cells, and olfactory/vomeronasal cells were cultured in DMEM/F12 (Invitrogen) supplemented 1% L-glutamine (Invitrogen) and D-(+)-glucose (final concentration 1%) at 37°C with 5% CO_2_ for 24 h in the presence or absence of mouse monoclonal anti-Sema3A (15 µg/ml) [57] neutralizing antibody (R&D Systems, MAB-1250) before processing for immunocytochemistry (control, *N* = 3 independent experiments, *n* = 146 cells; treated, *N* = 3 independent experiments, *n* = 143 cells). Anti-Sema3A binding in living cells was visualized using an AlexaFluor 568–conjugated anti-mouse antibody (1∶400; Invitrogen), and GFP using an anti-GFP chicken primary antibody (1∶1,000, ab13970, Abcam) and an AlexaFluor 488–conjugated anti-chicken secondary antibody (1∶400; Jackson Immunoresearch, West Grove, PA) in 4% paraformaldehyde fixed cultures.

Quantification of GnRH fiber length was performed on digitized photomicrographs using the NeuronJ plugin of ImageJ software (National Institutes of Health); 10–20 pictures were taken for each culture well, and a total of 200 cells for each treatment condition were analyzed. Twelve embryos were used for the control group and 13 for the treatment group. All experiments used primary cultures generated from different individuals on multiple culture dates. Data are presented as means ± SEM. For comparison between the two groups, a two-tailed unpaired Student's *t* test was used. Normality of the data was tested with the Shapiro-Wilk test.

### Cell Lines

COS-7 and SVEC4-10 cells (ATCC) were grown in a monolayer at 37°C in a 5% CO_2_ atmosphere, in DMEM (Life Technologies, Inc.) containing 1 mM sodium pyruvate, 2 mM glutamine (Life Technologies, Inc.), 100 µg/ml streptomycin, 100 U/ml penicillin, and 4,500 mg glucose (ICN Biomedicals, Inc.), supplemented with 10% FBS (Invitrogen). The medium was replaced at 2-d intervals. Subconfluent cells were routinely harvested by trypsinization and seeded onto 58 cm^2^ dishes (100,000 cells). For all experiments, only cells within six passages were used.

GnV-3 cells are one of 11 clones of GnRH-expressing cells obtained by the conditional immortalization of cultured adult rat hypothalamic cells [58]. GnV-3 cells express markers of well-differentiated neurons and do not express markers of glial cells [59]. Cells were grown in Proliferation medium consisting of Neurobasal A medium with B27 supplement (20 µl/ml, Invitrogen-Gibco), PSN (1×, Invitrogen), Glutamax I (Invitrogen), doxycycline hydrochloride (0.5 µg/ml, Sigma), FBS (10 µl/ml, Biological Industries), and βFGF (5 ng/ml, Invitrogen). Doxycycline promotes the proliferation of these conditionally immortalized cells. To induce the differentiation of GnV-3 cells, the culture medium was replaced by differentiation medium (containing Neurobasal A, B27 supplement, PSN, Glutamax I, and βFGF).

### In Vitro Cell Transfection

COS-7 cells were transiently transfected using the fast-forward protocol. Briefly, a 58 cm^2^ subconfluent dish was split into four dishes in OptiMEM medium (Invitrogen) about 1 h before high-efficiency liposome transfection (Lipofectamine 2000, Invitrogen). Each dish was transfected with 2–4 µg of DNA construct (full-length human Semaphorin 3A-myc cDNA plasmid, 65 kDa truncated human Semaphorin 3A-myc cDNA plasmid, and empty vector for control). The latter construct was generated by site-directed mutagenesis using a QuickChange II XL site-directed mutagenesis kit (Agilent Technologies) to introduce a Stop codon after the conserved Arginine residue 555, corresponding to the furin cleavage site.

### Cell Aggregates

Three-dimensional matrix assays were performed by co-culturing GnV-3 cell aggregates with ME explants dissected from adult female rats as described above. In another set of experiments, GnV-3 cell aggregates were co-cultured with COS-7 cells transfected either with full-length Sema3A (Sema3A-FL) or 65 kDa Sema3A, or with the control vector, as previously described [60].

For the aggregates, cells were collected by trypsinization, resuspended in 5 µl of growth-factor-free Matrigel (BD Biosciences, San Jose, CA) (10^6^ cells/ml for both GnV-3 and COS-7), and placed on the lid of a culture dish. As cell aggregates were formed in the droplets, they were plated onto Millicell inserts coated with growth-factor free Matrigel (Millipore) and maintained in culture for 72 h. Cultures were grown for 2–3 d in Neurobasal medium containing B27 and gentamycin before staining. To test whether Sema3A acts on GnRH-1 processes in an Nrp1-dependent fashion, a rat-Nrp1-neutralizing antibody (1 µg/ml, R&D Systems, AF566) was added to the growth medium of the co-cultures. The following day, the cultures were fixed with 4% paraformaldehyde in 0.01 M phosphate buffer, pH 7.4, and permeabilized with 0.3% Triton X-100 (Sigma) for 1 h at room temperature. Finally, they were stained with Alexa 568–X phalloidin (Molecular Probes, Eugene, OR) for 45 min at 37°C before image analysis. Quantification of GnV3 fiber growth was performed on digitized photomicrographs using the NeuronJ plugin of ImageJ software (National Institutes of Health).

### Image Analysis

For confocal observations and analyses, an inverted laser scanning Axio observer microscope (LSM 710, Zeiss) with EC Plan NeoFluor 10×/0.3 NA, 20×/0.5 NA, and 40×/1.3 NA (Zeiss) objectives was used (Imaging Core Facility of IFR114 of the University of Lille 2, France). ImageJ (National Institutes of Health, Bethesda, MD) and Photoshop CS5 (Adobe Systems, San Jose, CA) were used to process, adjust, and merge the photomontages.

### Intracerebral Infusion of Nrp1- or Sema3A-Neutralizing Antibodies

To determine the importance of Nrp1 in the central control of reproductive function, *in vivo* experiments were performed to neutralize Nrp1 receptor-ligand interactions within the ME. Anti- Nrp1 or -Sema3A IgGs (R&D Systems) were chronically infused into the ME (bregma −3.6 mm, 9.5 mm depth from the skull surface) [61] through a stereotaxically implanted infusion cannula (Plastics One, Roanoke, VA) connected to subcutaneously implanted osmotic minipumps (model 1007D; Alzet, Palo Alto, CA). The pump had a flow rate of 0.5 µl/h and a capacity of 100 µl, resulting in a delivery period of 7 d. Each pump was loaded with sterile DPBS (Invitrogen) containing the Nrp1-neutralizing antibody (0.25 µg/µl final) or no antibody. After connection to the infusion device and overnight priming in 0.9% NaCl at 37°C, the assembly was implanted into cycling 190–200 g female rats with regular estrous cycles. Estrous cycles were monitored before and after surgery.

Following infusion for 7 d, animals were killed to assess the implantation site of the cannula and check for exhaustion of the infused solution. MEs from Nrp1 and PBS-infused animals were collected, snap frozen in dry ice, and stored at −80°C. To determine whether the infused Nrp1 antibodies actually targeted the ME and bound endogenous Nrp1, protein extracts from MEs were prepared and subjected to immunoprecipitation using Nrp1 antibodies as described above. The sepharose beads from the preclearing step allowed IgGs to be collected from the MEs. The beads were then separated by centrifugation, washed twice with ice-cold lysis buffer, and boiled for 5 min in 50 µl of 2× NuPAGE LDS sample buffer (Invitrogen). After centrifugation, the supernatants were analyzed by Western blotting for Nrp1. Anti-Sema3A-treated animals and their PBS-treated controls were perfused transcardially with fixative and processed for immunohistochemistry as described above. AlexaFluor 488–conjugated anti-mouse antibodies (1∶400) were used to detect the binding of the intracranially infused Sema3A antibodies *in situ*; vascular endothelial cells were visualized using TRITC-conjugated *Bandeiraea simplicifolia* lectin.

### TAT-Cre Delivery

A TAT-Cre fusion protein produced as detailed previously [35] was injected into the tail vein (40 µl at 2.1 mg/ml) of mice 1 wk before they were placed for 62 h in a cage that had previously held a sexually experienced male to induce, a protocol used to induce a preovulatory surge in adult virgin mice [37].

### Quantitative RT-PCR Analyses from Mouse Hypothalamic Explants

For *Sema3a* gene expression analysis, mRNAs obtained from microdissected ME and mediobasal hypothalamus explants were reverse transcribed using SuperScript III Reverse Transcriptase (Life Technologies). Real-time PCR was carried out on Applied Biosystems 7900HT Fast Real-Time PCR System using the SEMA3A (Sema3a_Mm00436469_m1) exon-boundary-specific TaqMan Gene Expression Assays (Applied Biosystems).

### Plasma LH Assay

Plasma LH was measured using a Rodent LH ELISA kit (Endocrine Technologies, Newark, CA) with a sensitivity of 0.03 ng/ml and 7% intra-assay and 10% inter-assay coefficients of variance.

### Statistics

All analyses were performed using Prism 5 (GraphPad Software) and assessed for normality (Shapiro-Wilk test) and variance, when appropriate. Sample sizes were chosen according to the standard practice in the field. Before statistical analysis, percentages were subjected to arc-sine transformation to convert them from a binomial to a normal distribution [62]. Data were compared by a two-tailed unpaired Student's *t* test, one-way ANOVA for multiple comparisons, or two-way repeated-measures ANOVA. A Tukey's *post hoc* test was performed when appropriate. The significance level was set at *p*<0.05. Data groups are indicated as mean ± SEM. The number of biologically independent experiments, *p* values, and degrees of freedom are indicated in the figure legends.

## Supporting Information

Figure S1 **RT-PCR analysis of Sema3A, PV1, β3-Tubulin (β3Tub), DARPP-32, TSH, and GAPDH transcripts (gel image) in PV1-positive (PV1-pos) and PV1-negative cells isolated by FACS from the ME of adult female rats.**
(TIF)

Figure S2 **Cultured mouse endothelial cells.** (A) Immunopurified endothelial cells of the ME cultured *in vitro* are labeled with TRITC-conjugated Bandeiraea simplicifolia lectin (BslI, red) and exhibit PV1 immunoreactivity (green). Scale bar, 20 µm. (B) SVEC4–10 mouse endothelial cells mainly release p65 Sema3A, the proteolytic product of a 95 kDa precursor, released by furin cleavage.
(TIF)

Figure S3 **Nrp1 is expressed by vascular endothelial cells of the ME.** Confocal images showing the localization of Nrp1 immunoreactivity (green) in coronal sections of the ME of adult female rats. Vascular endothelial cells are labeled with TRITC-conjugated Bandeiraea simplicifolia lectin (BslI, red). Note that in addition to its abundance in the parenchyma of the ME (top panels), Nrp1 immunoreactivity is also found in endothelial cells of portal blood capillaries (bottom panels, arrows). Bottom panels are high-magnification images of the framed areas shown in (A). Scale bars, 100 µm in top and 50 µm in bottom panels.
(TIF)

Figure S4 **Nrp1- and Sema3A-neutralizing antibodies were efficiently delivered into the ME of adult female rats.** (A) Immunoprecipitation (IPP) and immunoblot (IB) analyses showing Nrp1 targeting by Nrp1-neutralizing antibodies (Nrp1-Ab) infused into the ME. At the end of the infusion period, MEs were microdissected, proteins extracted, and equal amounts of proteins incubated with protein A-sepharose beads to precipitate free IgGs (preclearing). The precipitated proteins were subjected to Western blotting and the supernatant used for immunoprecipitation. Note that in protein extracts from PBS-infused animals, no Nrp1 immunoreactivity was seen in the precleared fraction of the samples, while a strong Nrp1 immunoreactive signal was obtained after immunoprecipitation. In contrast, in protein extracts from the ME of Nrp1-Ab-treated rats, Nrp1 immunoreactivity was found in both the precleared and immunoprecipitated fractions of samples, showing the proportion of endogenous Nrp1 receptors bound and unbound by the infused antibody, respectively. SB, well loaded with sample buffer only. (B) Representative images showing the binding of intracranially infused Sema3A-neutralizing antibodies (green fluorescence, Alexa 488) in the ME. Arrows show the injection site. Note that the Sema3A-neutralizing antibodies selectively target the capillary zone of the ME, in which vascular endothelial cells are labeled with TRITC-conjugated Bandeiraea simplicifolia lectin (BSLI), and the surrounding nervous parenchyma. ARH, arcuate nucleus of the hypothalamus (ARH). (C) Representative Western blot image of conditioned media from transfected COS-7 cells producing the 65 kDa or the 95 kDa full-length Sema3A proteins.
(JPG)

Figure S5 **Full-length photographs of each of the Western blots presented in** **Figure 1F** **,** **Figure 1G** **,** **Figure 1H** **, Figure S1, and Figure S3 (IB, immunoblot).**
(TIF)

## Abbreviations

DARPP-32: dopamine- and cyclic-AMP–regulated phosphoprotein of molecular weight 32,000
E_2_: 17β-estradiol 3-benzoate
ER: estrogen receptor
GAP-43: growth-associated protein-43
GFP: green fluorescent protein
GnRH: gonadotropin-releasing hormone; MBH, mediobasal hypothalamus; ME, hypothalamic median eminence
Nrp1: neuropilin-1
OVX: ovariectomized
P: progesterone
PV1: plasmalemmal vesicle-associated protein 1
Sema3A: semaphorin 3A
TSH: thyroid-stimulating hormone
