# Supplementary figures and images for: Brain Endothelial Cells Control Fertility through Ovarian-Steroid–Dependent Release of Semaphorin 3A
Source: PLoS Biol. 2014 Mar 11;12(3):e1001808. doi: 10.1371/journal.pbio.1001808 (PMC3949669; doi:10.1371/journal.pbio.1001808)

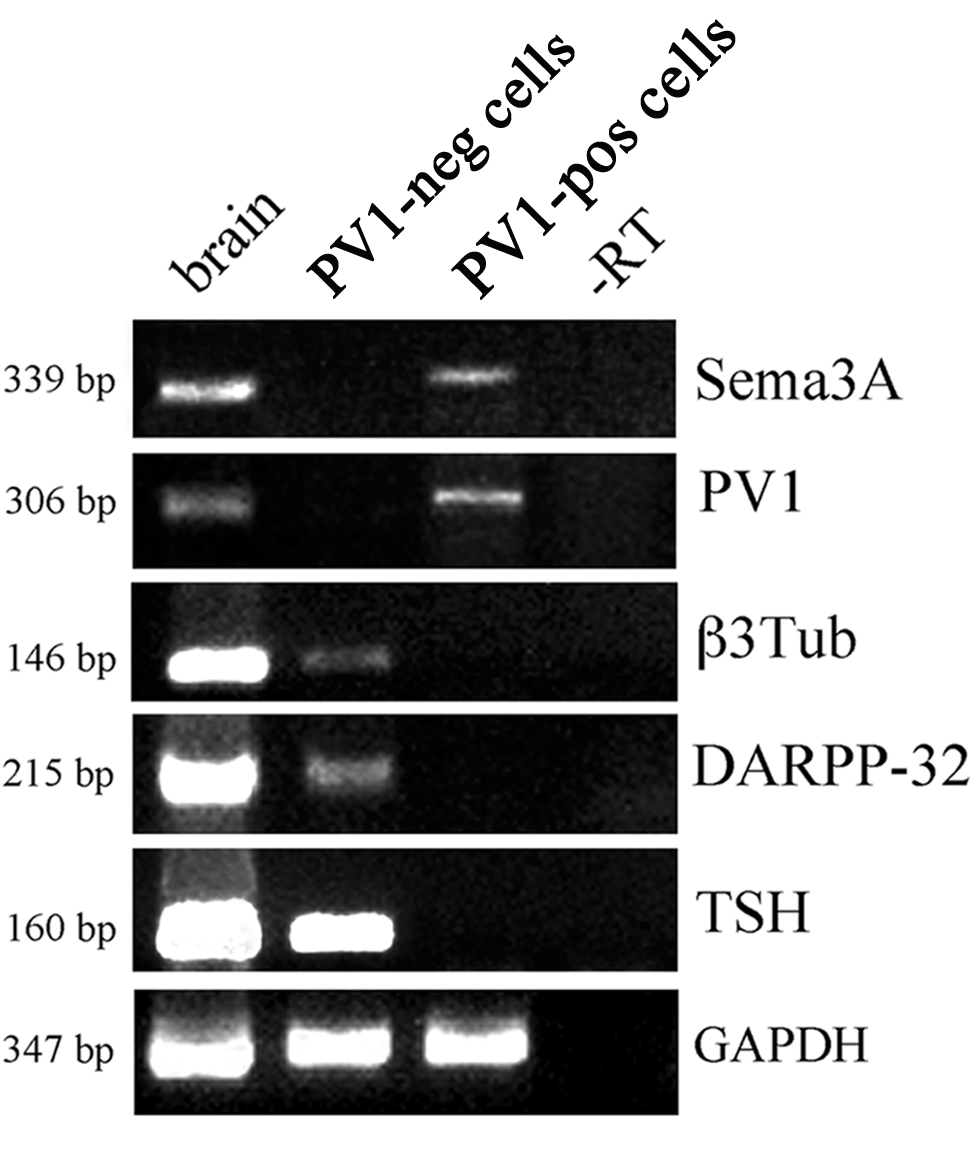

Supplement: Figure S1 — RT-PCR analysis of Sema3A, PV1, β3-Tubulin (β3Tub), DARPP-32, TSH, and GAPDH transcripts (gel image) in PV1-positive (PV1-pos) and PV1-negative cells isolated by FACS from the ME of adult female rats. (TIF) [file pbio.1001808.s001.tif]

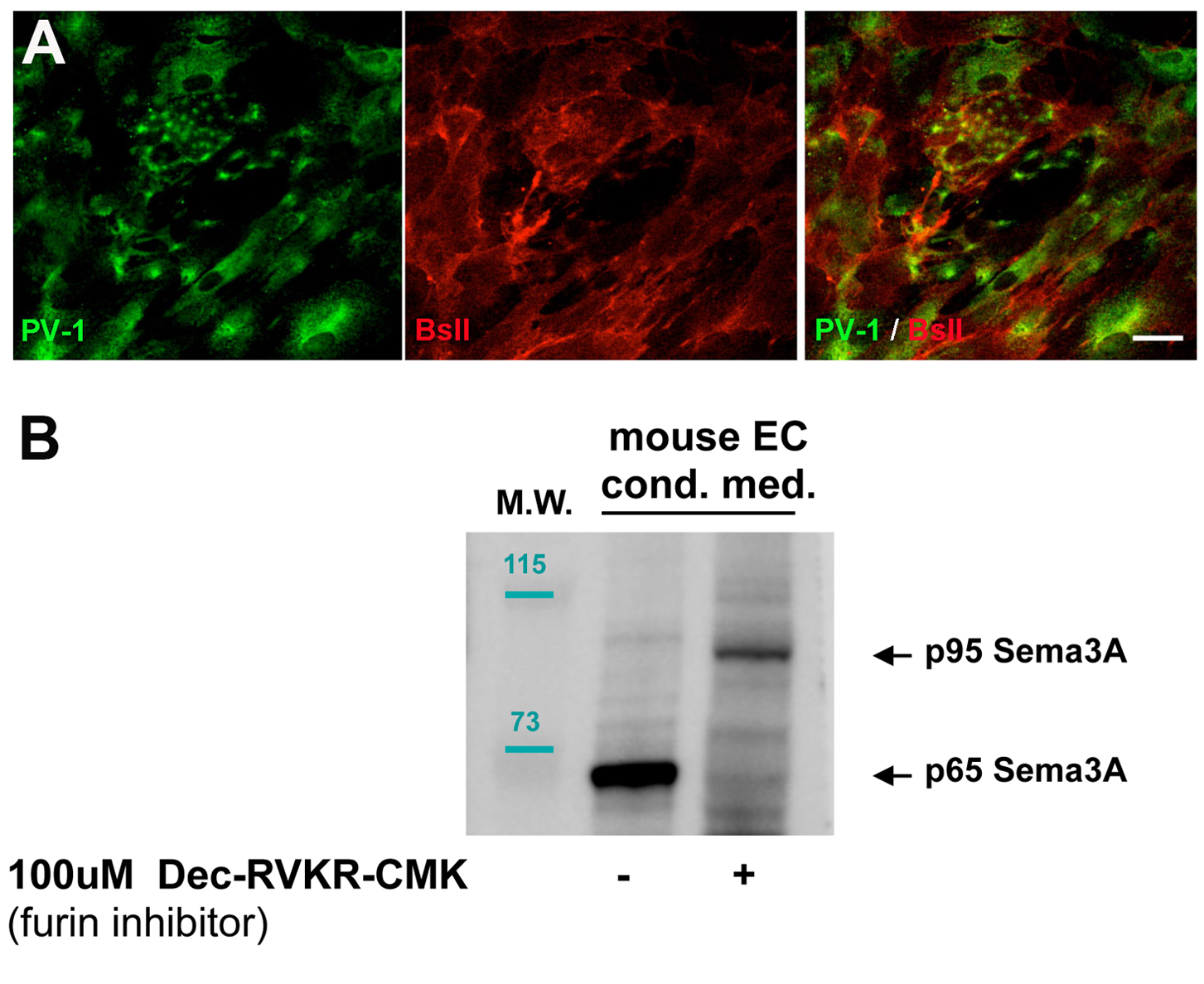

Supplement: Figure S2 — Cultured mouse endothelial cells. (A) Immunopurified endothelial cells of the ME cultured in vitro are labeled with TRITC-conjugated Bandeiraea simplicifolia lectin (BslI, red) and exhibit PV1 immunoreactivity (green). Scale bar, 20 µm. (B) SVEC4–10 mouse endothelial cells mainly release p65 Sema3A, the proteolytic product of a 95 kDa precursor, released by furin cleavage. (TIF) [file pbio.1001808.s002.tif]

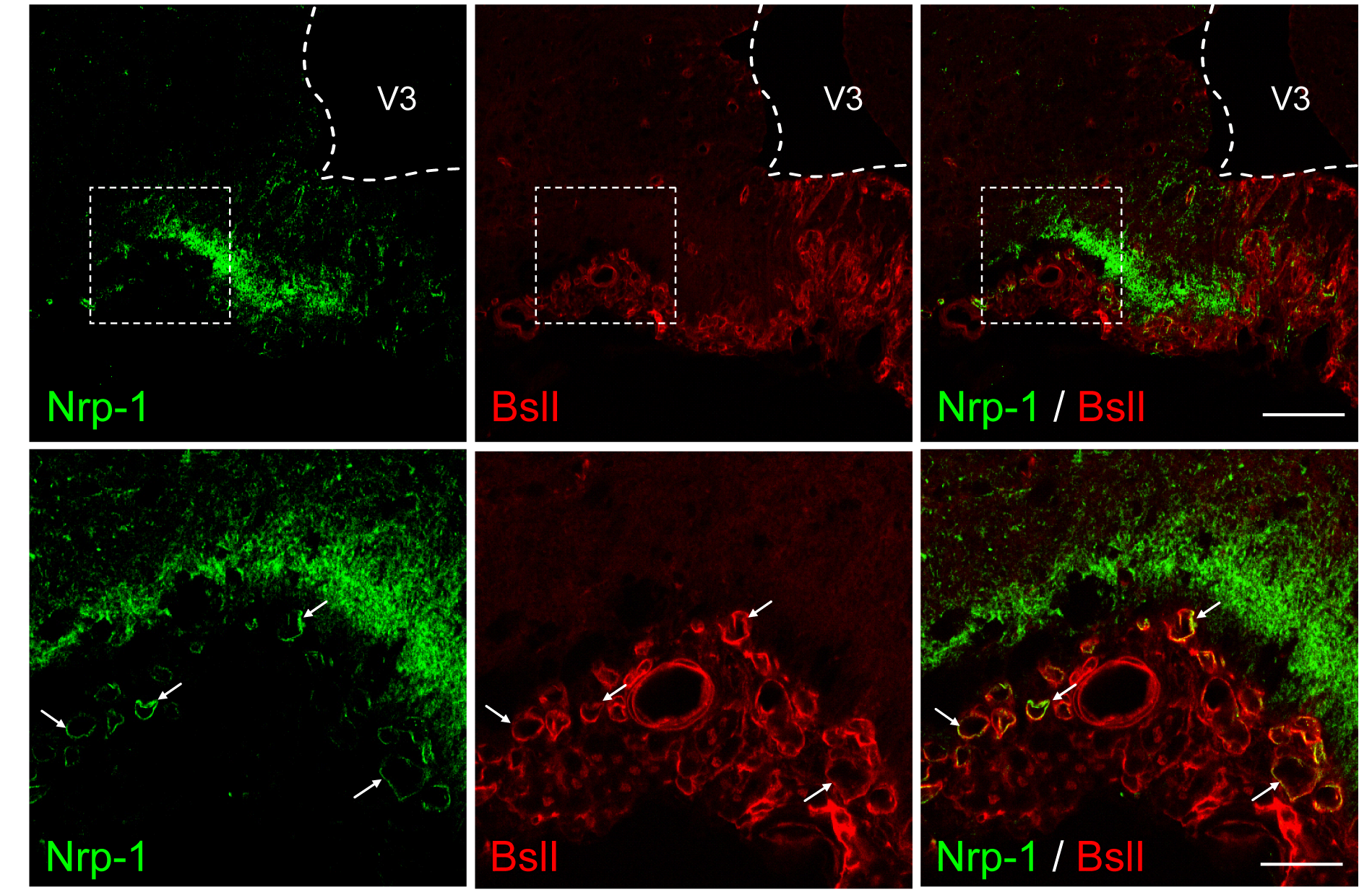

Supplement: Figure S3 — Nrp1 is expressed by vascular endothelial cells of the ME. Confocal images showing the localization of Nrp1 immunoreactivity (green) in coronal sections of the ME of adult female rats. Vascular endothelial cells are labeled with TRITC-conjugated Bandeiraea simplicifolia lectin (BslI, red). Note that in addition to its abundance in the parenchyma of the ME (top panels), Nrp1 immunoreactivity is also found in endothelial cells of portal blood capillaries (bottom panels, arrows). Bottom panels are high-magnification images of the framed areas shown in (A). Scale bars, 100 µm in top and 50 µm in bottom panels. (TIF) [file pbio.1001808.s003.tif]

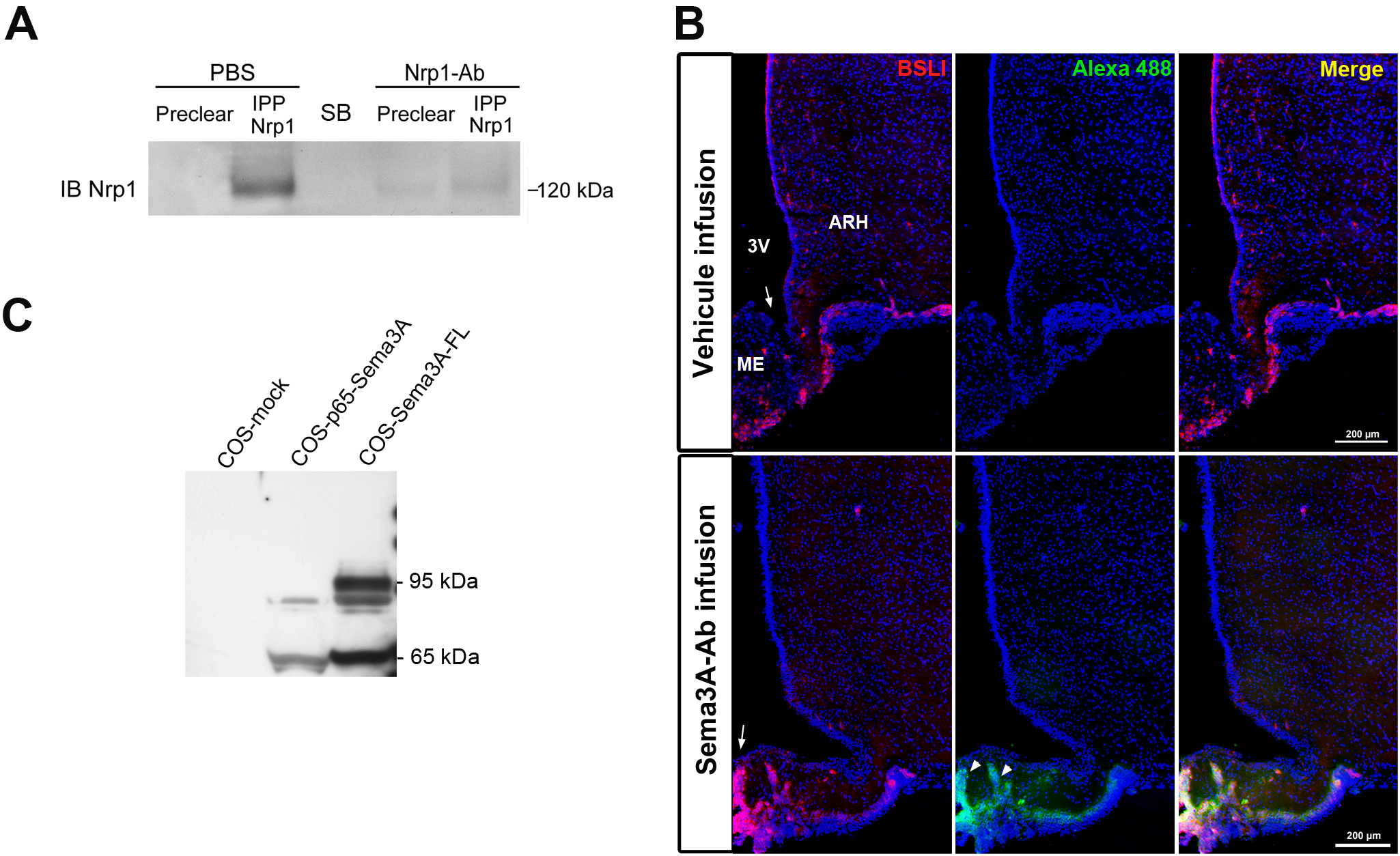

Supplement: Figure S4 — Nrp1- and Sema3A-neutralizing antibodies were efficiently delivered into the ME of adult female rats. (A) Immunoprecipitation (IPP) and immunoblot (IB) analyses showing Nrp1 targeting by Nrp1-neutralizing antibodies (Nrp1-Ab) infused into the ME. At the end of the infusion period, MEs were microdissected, proteins extracted, and equal amounts of proteins incubated with protein A-sepharose beads to precipitate free IgGs (preclearing). The precipitated proteins were subjected to Western blotting and the supernatant used for immunoprecipitation. Note that in protein extracts from PBS-infused animals, no Nrp1 immunoreactivity was seen in the precleared fraction of the samples, while a strong Nrp1 immunoreactive signal was obtained after immunoprecipitation. In contrast, in protein extracts from the ME of Nrp1-Ab-treated rats, Nrp1 immunoreactivity was found in both the precleared and immunoprecipitated fractions of samples, showing the proportion of endogenous Nrp1 receptors bound and unbound by the infused antibody, respectively. SB, well loaded with sample buffer only. (B) Representative images showing the binding of intracranially infused Sema3A-neutralizing antibodies (green fluorescence, Alexa 488) in the ME. Arrows show the injection site. Note that the Sema3A-neutralizing antibodies selectively target the capillary zone of the ME, in which vascular endothelial cells are labeled with TRITC-conjugated Bandeiraea simplicifolia lectin (BSLI), and the surrounding nervous parenchyma. ARH, arcuate nucleus of the hypothalamus (ARH). (C) Representative Western blot image of conditioned media from transfected COS-7 cells producing the 65 kDa or the 95 kDa full-length Sema3A proteins. (JPG) [file pbio.1001808.s004.jpg]

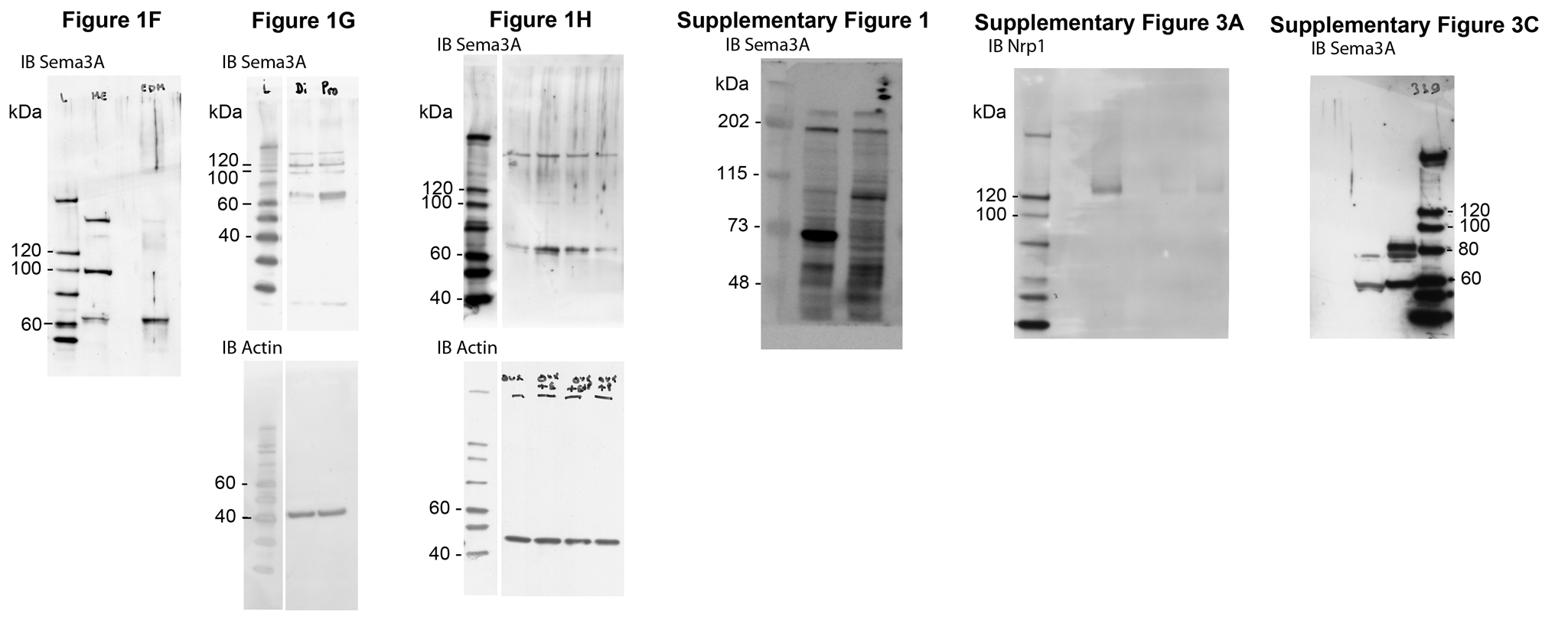

Supplement: Figure S5 — Full-length photographs of each of the Western blots presented in Figure 1F , Figure 1G , Figure 1H , Figure S1, and Figure S3 (IB, immunoblot). (TIF) [file pbio.1001808.s005.tif]
